# The Lymphatic Endothelial mCLCA1 Antibody Induces Proliferation and Growth of Lymph Node Lymphatic Sinuses

**DOI:** 10.1371/journal.pone.0156079

**Published:** 2016-05-25

**Authors:** Kimberly L. Jordan-Williams, Neela Ramanujam, Andrew G. Farr, Alanna Ruddell

**Affiliations:** 1 Department of Comparative Medicine, University of Washington, Seattle, WA, United States of America; 2 Fred Hutchinson Cancer Research Center, Seattle, WA, United States of America; 3 Department of Biological Structure, University of Washington, Seattle, WA, United States of America; University of Tokyo, JAPAN

## Abstract

Lymphocyte- and leukocyte-mediated lymph node (LN) lymphatic sinus growth (lymphangiogenesis) is involved in immune responses and in diseases including cancer and arthritis. We previously discovered a 10.1.1 Ab that recognizes the lymphatic endothelial cell (LEC) surface protein mCLCA1, which is an interacting partner for LFA1 and Mac-1 that mediates lymphocyte adhesion to LECs. Here, we show that 10.1.1 Ab treatment specifically induces LEC proliferation, and influences migration and adhesion *in vitro*. Functional testing by injection of mice with 10.1.1 Ab but not control hamster Abs identified rapid induction of LN LEC proliferation and extensive lymphangiogenesis within 23 h. BrdU pulse-chase analysis demonstrated incorporation of proliferating LYVE-1-positive LEC into the growing medullary lymphatic sinuses. The 10.1.1 Ab-induced LN remodeling involved coordinate increases in LECs and also blood endothelial cells, fibroblastic reticular cells, and double negative stroma, as is observed during the LN response to inflammation. 10.1.1 Ab-induced lymphangiogenesis was restricted to LNs, as mCLCA1-expressing lymphatic vessels of the jejunum and dermis were unaffected by 23 h 10.1.1 Ab treatment. These findings demonstrate that 10.1.1 Ab rapidly and specifically induces proliferation and growth of LN lymphatic sinuses and stroma, suggesting a key role of mCLCA1 in coordinating LN remodeling during immune responses.

## Introduction

The lymphatic system delivers fluid and molecules from tissues to lymph nodes (LNs) to allow for the surveillance of self and foreign antigen[1]. Recent studies suggest that the lymphatic sinuses within LNs can regulate the generation of a productive immune response, and that they can also promote tolerance of self antigen [2–4]. LNs undergo extensive architectural remodeling during immune responses involving expansion of lymphatic sinuses (lymphangiogenesis) accompanied by increases in other stromal components [5–8]. LN stromal cells include the lymphatic endothelial cells (LECs) that line the lymphatic sinuses, blood endothelial cells (BECs) that make up the high endothelial venules and capillaries, the fibroblast reticular cells (FRCs) that provide scaffolding for lymphocyte migration within the LN, and double negative (DN) cells that make up the remainder of the LN stromal cell compartment [9, 10].

LN lymphangiogenesis is a feature of cancer, autoimmune disease, and inflammation [11–13]. In cancer, LN lymphangiogenesis predicts and promotes tumor metastasis to the draining LN [14, 15]. Expansion of the medullary and cortical lymphatic sinuses is B cell-dependent and restricted locally to the tumor-draining LN [16]. LN lymphangiogenesis involving B cell accumulation has also been identified in inflammation [17] and arthritis [18, 19], and is associated with disease resolution. Taken together, these findings suggest that lymphocyte interactions with lymphatic sinuses are important for LN lymphangiogenesis and the outcome of normal or pathological immune responses.

In response to inflammation, LN lymphatic sinus growth promotes the generation of a functional immune response via increased dendritic cell (DC) trafficking from tissue to the draining lymph node [5]. Within 3 to 5 days of the initiation of inflammation, lymphangiogenesis begins in the LN and has been shown to be B cell-dependent [5, 8]. Other studies have provided evidence that DCs are important for the initial phase of the LN LEC and stromal response to inflammation, while later phases of LN remodeling are T cell- and B cell-dependent [6, 20]. This lymphangiogenesis could at least in part involve VEGFR signaling, as LN VEGF-A levels are sometimes elevated during immune responses [12, 17, 20, 21]. However VEGFR2 and VEGFR3 blocking Abs together only partially inhibited inflammatory LN lymphatic sinus growth [5, 17, 20], suggesting that additional signaling pathways are involved in regulation of lymphangiogenesis.

LN remodeling during immune responses involves not only LECs, but also a coordinated expansion of BECs, FRCs, and DN cells in the LN [6, 8, 20]. The expansion of LN stroma can be stimulated by IL-1β produced by CD11c+ cells [22], by IL-7 produced by LECs [23], and by lymphotoxin produced by lymphocytes [7, 8]. These findings suggest that multiple signaling pathways can promote different aspects of LN remodeling during immune responses. One consistent feature is the development of lymphatic sinus growth coordinated by lymphocytes and leukocytes, although little is known yet about how this lymphangiogenesis is achieved. Peripheral lymphatic vessel growth can involve LEC proliferation and migration [24] as well as recruitment of LYVE-1-expressing CD11b+ macrophages into growing lymphatic vessels [25–27]. Bone marrow progenitors also may contribute to postnatal lymphatic vessel growth, although it is not known whether these cells are macrophage precursors or if they represent some other type of progenitor [28, 29].

We identified a 10.1.1 hamster monoclonal Ab which recognizes murine chloride channel calcium-activated 1 (mCLCA1) protein as a surface marker of LECs [12, 30]. Although sequence comparisons initially suggested mCLCA1 function as a regulator of chloride channel function [31], this was not found to be the case [32, 33]. Unlike LYVE-1, which is down-regulated in response to inflammation [34], mCLCA1 expression on LECs is not impacted by TNF-α/IL-1β stimulation, suggesting that it is a useful marker of normal as well as inflamed LECs [30]. mCLCA1 is also expressed on collecting and initial lymphatic vessels [35] as well as on splenic red pulp stromal cells and thymic medullary stromal epithelial cells [12]. Our *in vitro* studies have identified a role for mCLCA1 as an interacting partner for LFA1 to mediate lymphocyte adhesion to LECs, as treatment of LECs with the 10.1.1 Ab significantly reduced lymphocyte adhesion [30]. Interestingly, 10.1.1 Ab inhibited lymphocyte adhesion to a greater extent than anti-ICAM1 Ab, suggesting that mCLCA1 is more important for lymphocyte-LEC interaction, in contrast to the LFA1-ICAM1 interactions that predominate in vascular endothelium [36]. These findings suggested that mCLCA1 functions in lymphatic/immune cell interactions. In this study, we investigated the function of mCLCA1 in LECs, and found that the 10.1.1 Ab activates lymphatic endothelium *in vitro*, and also rapidly induces LN lymphangiogenesis *in vivo*, suggesting a role for mCLCA1 in regulation of LN lymphatic sinus growth.

## Materials and Methods

### Mice, antibodies, and reagents

C57Bl/6J mice were obtained from The Jackson Laboratory (Bar Harbor, ME). Mice were housed in specific pathogen free facilities and all experimental methods involving animals carried out in strict accordance with the recommendations in the Guide for the Care and Use of Laboratory Animals of the National Institutes of Health. Protocols were approved by the University of Washington and Fred Hutchinson Center for Cancer Research Institutional Animal Care and Use Committees (4305–01). Mice were studied between 6–8 weeks of age. None of the mice became ill or died prior to the experimental endpoint. Mice did not require medical treatment or humane euthanasia before the end of studies as they did not show any signs of distress or discomfort during experiments.

The 10.1.1 [37] and 8.1.1 [38] hybridomas (Developmental Studies Hybridoma Bank) were grown and antibodies were purified from the supernatant by protein G-sepharose chromatography at the Fred Hutchinson Cancer Research Center Biological Production facility. Syrian Hamster IgG was obtained from Jackson ImmunoResearch Laboratories, West Grove, PA. The 10.1.1, 8.1.1, and Hamster IgG antibodies were tested for endotoxin levels using a ToxinSensor Chromogenic LAL Endotoxin Assay Kit (GenScript, Piscataway, NJ). Antibodies used for immunofluorescent staining and flow cytometry were: LYVE-1 or LYVE-1-AF488 (clone ALY7, eBiosciences), Ki67- or Ki67-eFluor 615 (clone SolA15, eBioscience), Prox1 (rabbit polyclonal, Millipore), CD11b-AF488 (clone M1/70, eBioscience), Podoplanin (clone 8.1.1, BioLegend), CD31-PE (clone 390, eBioscience), CD45-PE-Cy7 (clone 30-F11, eBioscience), anti-BrdU-FITC (BD Biosciences), goat anti-Syrian Hamster-AF568 (Invitrogen), goat anti-Rat-AF488 (Invitrogen), goat anti-Rabbit-AF568 (Invitrogen), anti-FITC-AF488 (Invitrogen), horseradish peroxidase anti-rat IgG (Southern Biotech).

### Immunostaining and image analysis

Tissues were harvested and embedded in OCT (Sakura, Finetek). Popliteal LNs were oriented under magnification before mounting in OCT, in order to obtain cross-sections through the red-colored medulla and the white-colored cortical follicles. Eight μm cryosections were fixed with 4% paraformaldehyde and immunostained as previously described [12]. Immunofluorescence slides were coverslipped with Prolong Gold Reagent (Life Technologies). For IHC staining, HRP conjugated secondary antibodies were detected with vector VIP reagent (Vector Laboratories), counterstained with Methyl Green (Vector Laboratories), and coverslipped with Permount (Fisher Scientific). Slides were imaged using a Nikon microscope, and images were processed using NIH ImageJ software.

Image quantification of lymphatic sinus area was performed using NIH ImageJ software. Images were thresholded to visualize LYVE-1 positive staining. The LN was outlined using the polygon drawing tool, and the percent lymphatic sinus area occupied by LYVE-1 staining was measured. Six mice from each treatment group were analyzed, using randomly chosen LNs. Three representative slides sampled through the entire popliteal LN of each mouse were analyzed and significance was determined using a Mann Whitney *U* test for unpaired samples using Prism (GraphPad Software).

### Flow cytometry

LN stroma was isolated by digesting LNs with Collagenase P (Roche) and Dispase I (Worthington Biochemical) as previously described [39, 40]. Single cell suspensions were stained with anti-CD45, anti-CD31, and anti-Podoplanin and data collected on a BD CantoII cytometer. Data was analyzed using FlowJo software (Treestar). Cell size was assessed by forward scatter profile. Significance was determined using a Mann Whitney *U* test for unpaired samples using Prism (GraphPad Software).

### *In vitro* proliferation assay

LNs were digested as described above [39, 40] or with Collagenase D (Worthington) and DNAse I (BD Biosciences) as described [2, 41]. The cells from each mouse were plated into a 4-well chamber slide (Lab-tek, Nunc) in DME (Invitrogen) plus 10% fetal calf serum (Hyclone). After 24 h, non-adherent cells were removed and the remaining stromal cells were cultured in the presence of 30 μg/ml 10.1.1 Ab or control Hamster IgG for 5 days. Cells were stained with anti-Prox1 and anti-Ki67 Abs to identify proliferating LECs. Cells in at least 6 random 20x fields from each chamber were counted. Six mice were analyzed for each Ab treatment. Significance was determined using a Wilcoxon Ranked Sum test for paired samples using Prism (GraphPad Software).

### Lymphatic endothelial tube formation assay

SV-LEC [42] were plated at a density of 2.5x10^5^ cells/well in a 12-well plate in DMEM containing 2% FBS. Cells were cultured for 16 h in the presence or absence of antibody or lymphocytes. For antibody treatment, cells were cultured in the presence of 30 μg/ml Hamster IgG or 10.1.1 Ab. For lymphocyte treatments, splenocytes were isolated [30], and were co-cultured with SV-LECs, at 1x10^6^ cells/ml. All cells were harvested after the 16 h treatment period using Trypsin. Cells were washed once in media to remove Trypsin and plated at 2x10^4^ SV-LEC cells/100ul on top of 50ul of pre-set Growth Factor Reduced Matrigel in a 96-well plate. Samples were incubated for 4 h followed by staining of cells with Calcein AM 8 μg/ml (BD Biosciences). Tubes were visualized at 4x magnification using a Nikon microscope. The percent area occupied by tubes was calculated using NIH ImageJ software and significance was determined using a Mann Whitney *U* test for unpaired samples using Prism (GraphPad Software).

### BrdU Pulse-Chase

Mice were injected intraperitoneally (i.p.) with 200 μg 10.1.1 Ab or control Hamster IgG at time 0 h. At 16 h post Ab injection, mice were injected i.p. with 1 mg BrdU (BD Biosciences). 2 h post BrdU injection, the pulse-chase cohort mice were injected i.p. with 5 mg Thymidine (Sigma). At the t = 2 h time point, the pulse cohort was euthanized by CO_2_ asphyxiation and tissues harvested. At 20 h after thymidine injection, the pulse-chase cohort was euthanized by CO_2_ asphyxiation and tissues harvested. Cryosections were generated, blocked in 10% goat serum (Sigma), and incubated in 2N HCl for 1 h to denature DNA prior to incubation with anti-BrdU Ab. Slides were stained with anti-BrdU-FITC antibody followed by anti-FITC-AF488 secondary. Slides were then fixed again in 4% paraformaldehyde, blocked with 10% goat serum, and stained with anti-LYVE-1 antibody followed by anti-Rat-AF568 secondary.

## Results

### 10.1.1 Ab induces LN LEC proliferation *in vitro*

**Fig 1:**
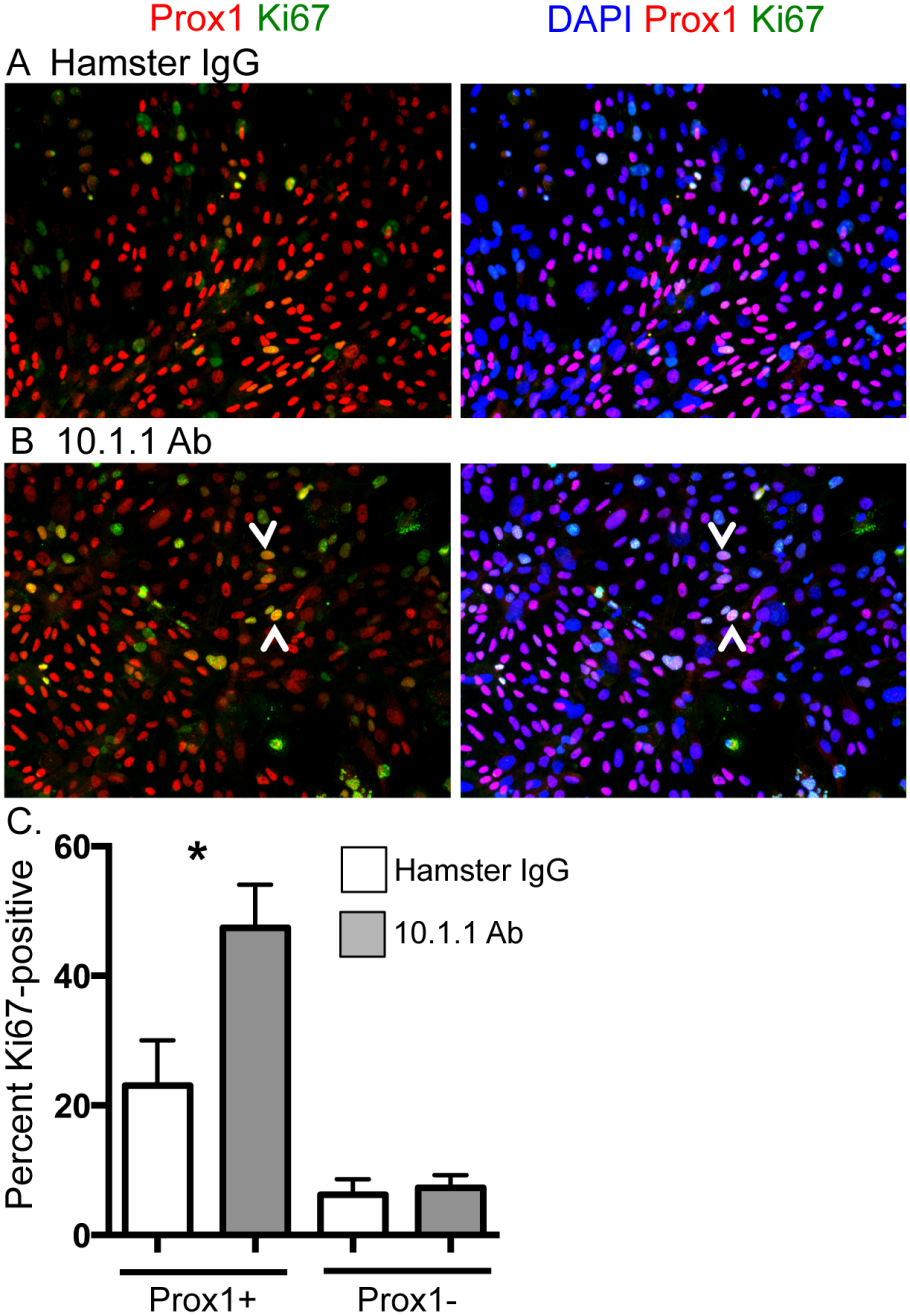
10.1.1 Ab induces proliferation of LECs *in vitro*. Pooled axillary, brachial, and inguinal LNs were enzymatically digested and plated into chamber slides. Cells were treated with antibody for 5 d and stained to identify proliferating LECs. A). Immunostaining of hamster IgG-treated cultures identifies Prox1+ LECs (red) and Ki67+ proliferating cells (green), and confirms nuclear location of Prox1 and Ki67 by blue nuclear DAPI staining (right panel). B). Immunostaining of 10.1.1 Ab-treated cultures identifies a number of Prox1+ and Ki67+ proliferating LECs (e.g. arrowheads, left panel), and confirms nuclear location of Prox1 and Ki67 by DAPI staining (arrowheads, right panel). C). The Ki67+Prox1+ or Ki67+Prox1- cells from 6 preparations were counted in five fields for each sample, and the percentage of each population was determined. 10.1.1 Ab-treated samples display increased proliferating LECs compared to control Hamster IgG-treated samples. Significance was determined using a Wilcoxon Ranked Sum test for paired samples. *: p<0.05. Standard errors are indicated.

The function of LEC mCLCA1 was first investigated by incubating 10.1.1 Ab with primary LN LECs *in vitro*. LN stromal cells were isolated from pooled LNs by collagenase digestion, and adherent cells were cultured on chamber slides either in the presence of 10.1.1 Ab or control Hamster IgG for 5 days. This isolation and culture method results in a mixed culture consisting of LECs, BECs, FRCs, and DN stromal cells [39, 40]. While the morphology of cells in these cultures was similar, mitotic figures were common in the 10.1.1 Ab-treated cultures, prompting development of an assay to measure proliferation. Slides were immunostained with Prox1 Ab to identify LECs expressing this transcription factor specifying lymphatic cell fate [43], and with anti-Ki67 to identify proliferating cells (Fig 1A and 1B). The 10.1.1 Ab increased proliferation of Prox1+ cells more than two-fold relative to Hamster IgG-treated cells (Fig 1C). In contrast, no change in proliferation of Prox1-negative stromal cells (non-LECs) was identified, demonstrating that the 10.1.1 Ab specifically induces proliferation of LECs and not other stromal cell types *in vitro*. This presumably is due to specific binding of the 10.1.1 Ab to LECs but not other stromal cell types, as our immunostaining [12], electron microscopy [12] and flow cytometry studies [22] have only identified 10.1.1 Ab expression on LECs.

### 10.1.1 Ab blocks LEC migration and adhesion *in vitro*

**Fig 2:**
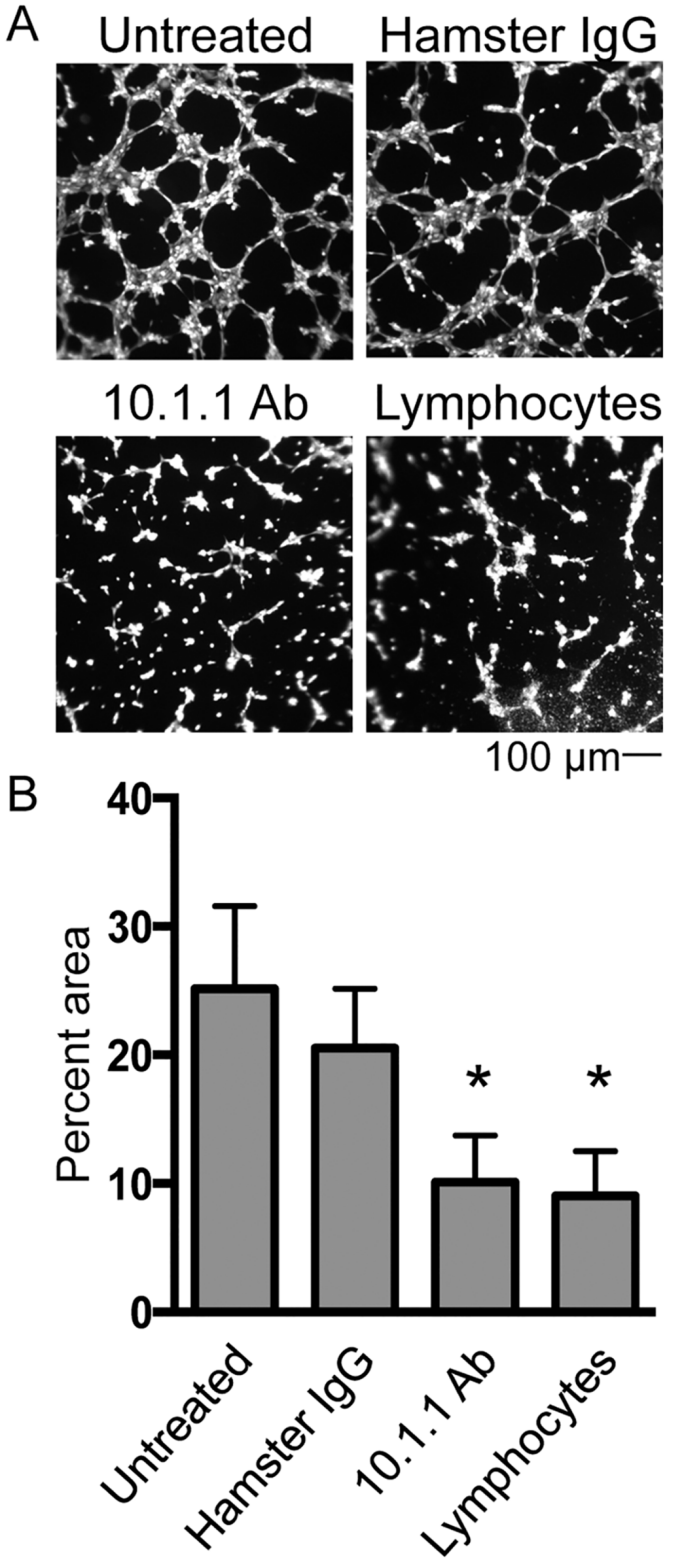
10.1.1 Ab or lymphocyte co-culture similarly block *in vitro* tube formation. SV-LECs were treated overnight with 30 μg/ml Hamster IgG or 10.1.1 Ab or co-cultured overnight with lymphocytes. Cells were trypsinized and plated on growth factor-reduced Matrigel at equal densities and allowed to form tubes. A). Cells were stained with Calcein AM and 4x images were analyzed. Scale bars are indicated. B). The percent area occupied by the tubes was quantified using Image J. 10.1.1 Ab treatment and lymphocyte co-culture similarly reduced tube formation potential of SV-LECs while Hamster IgG control Ab did not impact tube formation. Significance was determined using a Mann Whitney *U* test for unpaired samples. n = 5, * p<0.05.

A tube formation assay was then performed to test whether mCLCA1 ligation by 10.1.1 Ab influences LEC migration or adhesion. The SV40-transformed LEC cell line SV-LEC was used for these studies, as it readily grows in culture while retaining many aspects of LEC function [42]. Cultures were pre-treated with 10.1.1 Ab or control Hamster IgG and then plated onto growth factor-reduced Matrigel. In all conditions, SV-LECs were stained with Calcein AM to visualize tubes and also to confirm cell viability, in order to check for the possibility of decreased viability that could impact tube formation potential. No difference was observed whether phase contrast total cells or fluorescent Calcein M viable cell images were examined (data not shown), indicating that cell viability was not affected by these treatments. Cells treated with nonspecific Hamster IgG formed tubes similar to untreated cells, while cells incubated with the 10.1.1 Ab failed to form a tube network (Fig 2A), instead forming small clumps. Interestingly, SV-LECs treated with mouse lymphocytes also failed to form tubes, showing a disorganized pattern similar to that observed with 10.1.1 Ab treatment (Fig 2A).

The percent area occupied by the tube network was quantified for each condition using NIH ImageJ software. 10.1.1 Ab-treated and lymphocyte co-cultured SV-LEC both displayed a statistically significant 2- to 3- fold reduction in area compared to untreated or Hamster IgG treated control cells (Fig 2B), confirming that these LEC reproducibly failed to form tubes. These findings demonstrate that the 10.1.1 Ab mimics lymphocyte behavior to influence LEC migration and adhesion activity.

### The 10.1.1 Ab rapidly and specifically induces LN lymphangiogenesis

**Fig 3:**
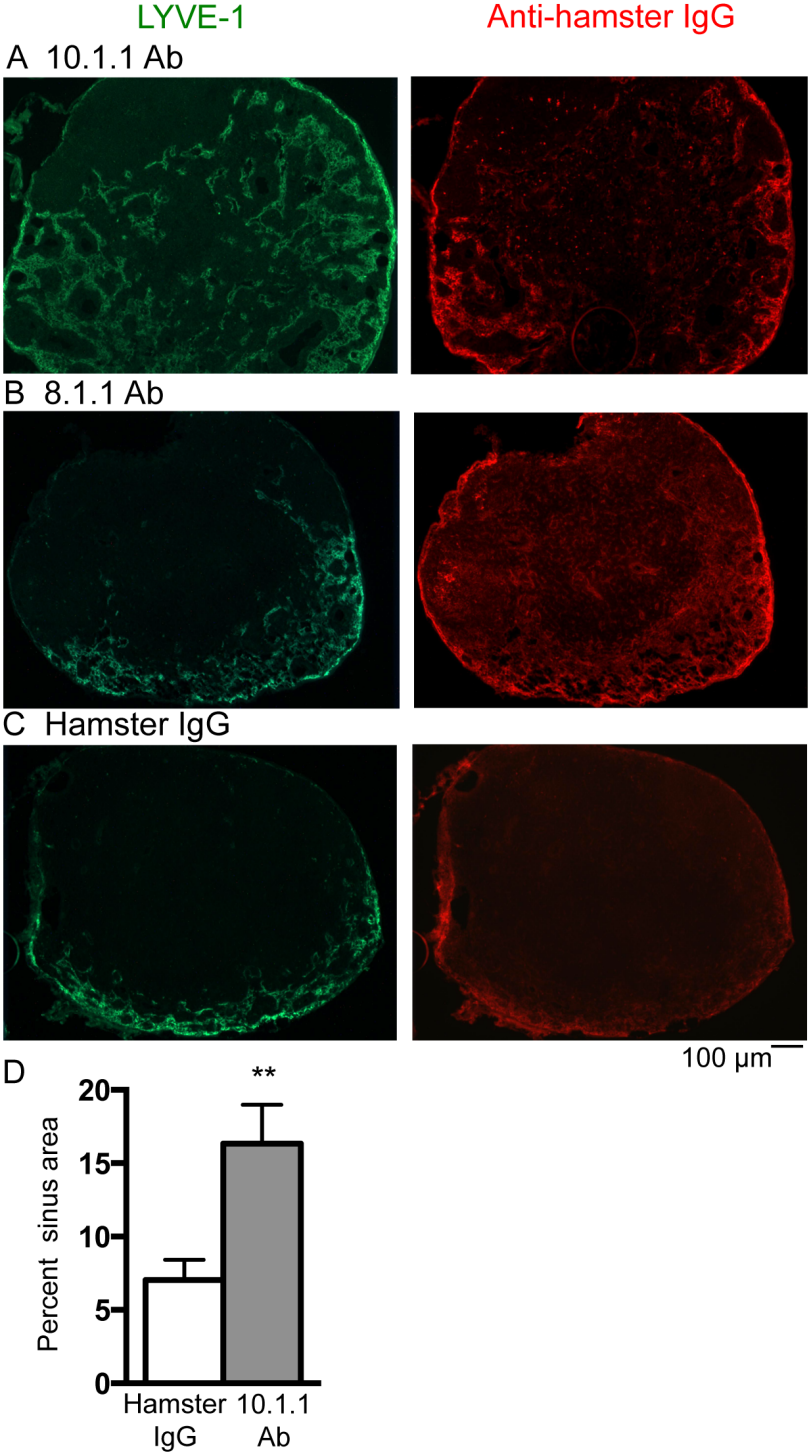
10.1.1 Ab injection rapidly induces expansion of lymph node medullary sinuses. A-C). Left panels: Popliteal LN cryosections were immunostained with LYVE-1 antibody to identify lymphatic sinuses. Lymphatic sinus area was greatly increased and extended from the medulla toward the cortex in 10.1.1 Ab-injected mice (A) compared to 8.1.1 Ab (B) or Hamster IgG -injected mice (C). A-C). Right panels: Sections were stained with anti-Hamster antibody to identify binding of the 10.1.1 Ab (A), 8.1.1 Ab (B), and Hamster IgG (C). 10.1.1 Ab binds to the medullary and cortical lymphatic sinuses in 10.1.1 Ab-injected mice (A, right panel). In 8.1.1 Ab-injected samples, 8.1.1 Ab is detected binding to the lymphatic sinuses and to individual cells (B, right panel). Hamster IgG-injected samples display non-specific antibody binding of anti-hamster secondary antibody (C, right panel). Representative images are shown with the medulla located at the bottom edge of each image. Scale bars are indicated. D). Images of popliteal LNs from Hamster IgG-injected and 10.1.1 Ab-injected mice stained with LYVE-1 were quantified using NIH Image J software. 10.1.1 Ab-injected mice exhibit increased lymphatic sinus area compared to control Hamster IgG-injected mice. Six mice randomly chosen from each treatment group with three representative sections each were analyzed. Significance was determined using a Mann Whitney *U* test for unpaired samples. **p<0.005. Standard errors are indicated.

To test whether the 10.1.1 Ab influences lymphatic endothelial functioning *in vivo*, mice were injected i.p. with 10.1.1 Ab, control 8.1.1 Ab (anti-podoplanin), or control Hamster IgG. LNs were analyzed 23 h later by immunostaining for the LYVE-1 LEC surface marker [44] to identify lymphatic sinuses in the popliteal LNs. LYVE-1 Ab immunostaining demonstrated extensive spread of the lymphatic sinuses from the medulla toward the cortex in LNs from 10.1.1 Ab-injected mice (Fig 3A, left panel), but not 8.1.1 Ab- (Fig 3B, left panel) or Hamster IgG- (Fig 3C, left panel) injected control mice. The 10.1.1 Ab-injected mice (N = 15) analyzed all demonstrated growth of medullary lymphatic sinuses, while the lymphatic sinuses of 8.1.1 Ab-injected mice (N = 6), or of Hamster IgG (N = 6) did not exhibit lymphatic sinus expansion. This growth occurred mainly within the medulla (S1A Fig), rather than in the cortical sinuses surrounding the B cell follicles, detected by DAPI staining (S1B Fig). While 10.1.1 Ab consistently induced medullary sinus growth, the extent of growth at 23 h was variable, covering approximately 1/3 (S1A Fig) to 2/3 of the LN area (Fig 3A), probably due to the rapid onset of this growth. Otherwise, the morphology of the lymphatic sinuses was similar in all of the Ab-injected mice. The increase in popliteal LN lymphatic sinus area induced by 10.1.1 Ab was quantified, and demonstrated an average 2.3-fold increase in LYVE-1 staining in 10.1.1 Ab-injected mice compared to Hamster IgG-injected mice (Fig 3D).

Immunostaining experiments were performed using anti-hamster secondary Ab to confirm binding of the various injected Abs to their respective LN targets. The 10.1.1 Ab bound to the cortical and medullary lymphatic sinuses (Fig 3A, right panel), indicating that the injected 10.1.1. Ab effectively and specifically binds to LN lymphatic sinuses. Interestingly, 10.1.1 Ab-induced lymphangiogenesis was restricted to the medullary sinuses (Fig 3A, left panel), suggesting that 10.1.1 Ab binding is necessary but not sufficient for lymphatic sinus growth. The 8.1.1 anti-podoplanin Ab efficiently bound to lymphatic sinuses and to individual cells distributed throughout the LN (Fig 3B, right panel) as expected [45], although this binding did not induce LEC growth, supporting the specific effects of 10.1.1 Ab LEC binding to induce LN lymphangiogenesis. Similarly, hamster IgG bound nonspecifically (Fig 3C, right panel), although it did not have any effect on LN architecture. These findings demonstrate that 10.1.1 Ab binding to LN LECs specifically induces LN lymphatic sinus growth, and that podoplanin or hamster Ab binding to other LEC antigens is not sufficient to activate LN lymphangiogenesis. Moreover, all of the Ab preparations contained similar endotoxin levels (Hamster IgG: 0.94 IU, 8.1.1 Ab: 0.74 IU, and 10.1.1 Ab: 0.8 IU), controlling for inclusion of this impurity in the antibody preparation. The 10.1.1 and 8.1.1 Abs were both generated in the same hybridoma screen [37, 38] and were purified at the same time in a clinical Ab production facility, further controlling for any other nonspecific components of these antibody preparations.

Our finding that the 10.1.1 Ab was the only reagent able to induce LN lymphangiogenesis, and that this LN remodeling is rapidly induced within 23 h suggests that the 10.1.1 Ab induces these changes by directly binding to LN LEC mCLCA1. The ability of the 10.1.1 Ab to induce LN lymphangiogenesis was not restricted to the popliteal LN, as axillary LNs also exhibited increased medullary lymphatic sinuses in 10.1.1 Ab-treated but not hamster IgG-treated mice (S2A Fig). This lymphatic sinus growth does not involve lymphocyte accumulation within the sinuses, as the diameter of sinuses was similar in LNs from 10.1.1 Ab- or hamster IgG-injected mice, and the cells within sinuses were consistently LYVE-1-positive (S2B Fig). Instead, the LN lymphangiogenesis in 10.1.1 Ab-injected mice involves increased numbers of lymphatic sinuses.

### The 10.1.1 Ab coordinately increases lymph node lymphatic endothelial and stromal cell content

**Fig 4:**
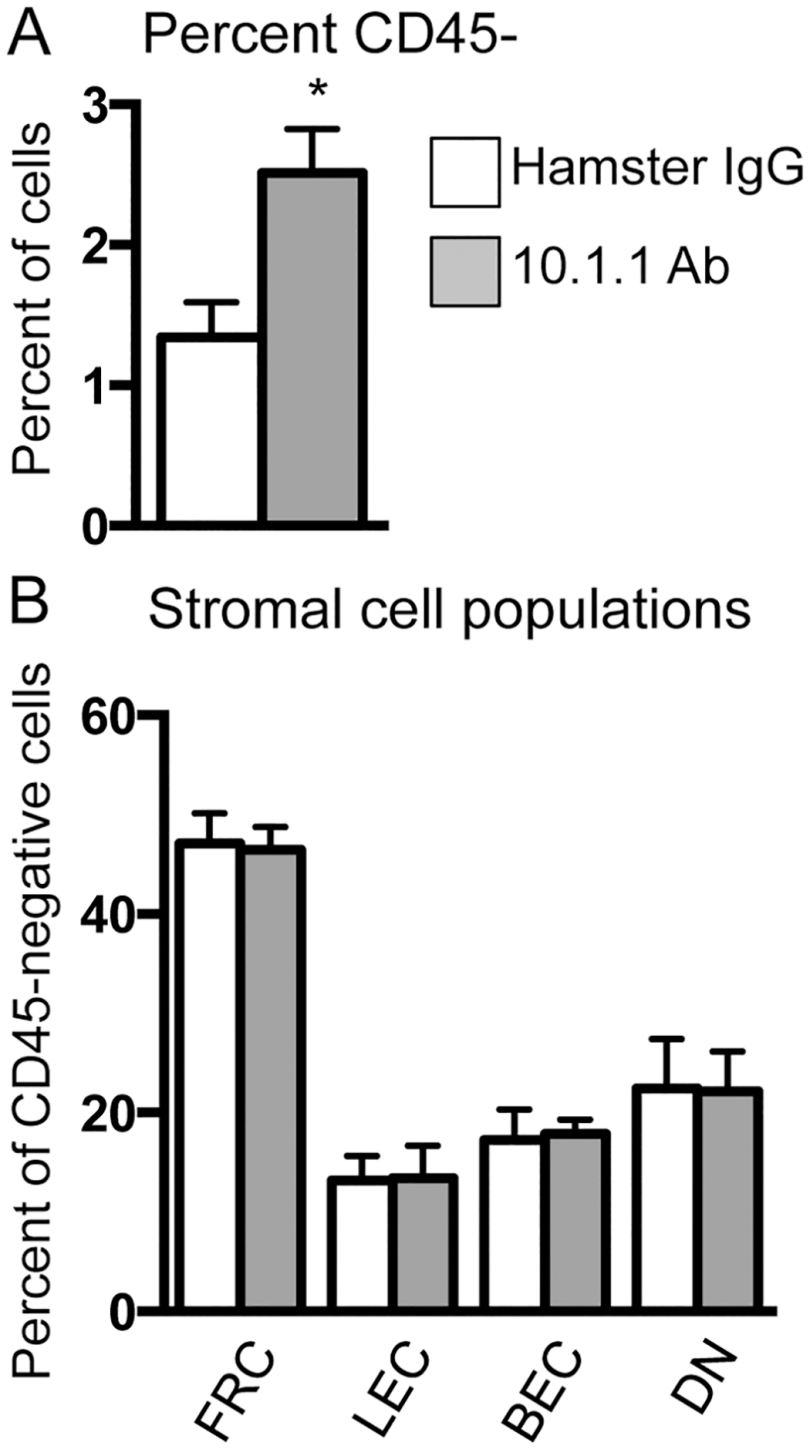
10.1.1 Ab induces coordinate accumulation of lymphatic endothelial and stromal cells. Pooled LNs were enzymatically digested and stained with CD45, CD31, and Podoplanin antibodies for flow cytometry analysis. Viable cells were gated as CD45- to detect all stroma and then further gated to identify LEC (CD31+ Podoplanin+), FRC (CD31- Podoplanin+), BEC (CD31+ Podoplanin-), and DN stromal cells (CD31- Podoplanin-). A). 10.1.1 Ab-injected mice display an increased percentage of increased CD45- cells. B). Populations are shown as a percent of CD45- cells. All cell types remain proportional in the 10.1.1 Ab-injected mice indicating a coordinate expansion of LN stromal cell populations in response to 10.1.1 Ab treatment. Significance was determined using a Mann Whitney *U* test for unpaired samples. n = 6, p<0.05. Standard errors are indicated.

The rapid 10.1.1 Ab-induced LN lymphangiogenesis *in vivo* could involve increased LEC proliferation, as the 10.1.1 Ab induced proliferation of LECs *in vitro*. To assess whether LN LEC representation increases in response to 10.1.1 Ab treatment, LNs were digested with collagenase and dispase to isolate stromal components including LEC, FRC, BEC, and DN stromal cells, and the cells were stained with CD45, CD31, and Podoplanin gp38 antibodies for analysis by flow cytometry [40], with examples shown in S3 Fig. 10.1.1 Ab-injected mice showed a more than 2-fold increase in the proportion of CD45- cells in LNs (Fig 4A). It was not possible to reliably quantify the absolute increase in LN LECs (Fig 4A), as they comprise a small percent of the total LN cells [40]. However, amongst the CD45- population, all stromal cell types (LEC, FRC, BEC, and DN) were present in the same proportions in the 10.1.1 Ab-treated and control Hamster IgG-treated mice (Fig 4B). These findings indicate that LN LEC abundance is increased by 10.1.1 Ab-treatment, and that BEC, FRC, and DN stroma also accumulate proportionately during this LN remodeling. As the 10.1.1 Ab binds specifically to LECs, and not to other stromal cell types [2, 12], the coordinate expansion of the other stromal cell types *in vivo* could somehow be linked to 10.1.1 Ab-induced lymphangiogenesis.

**Fig 5:**
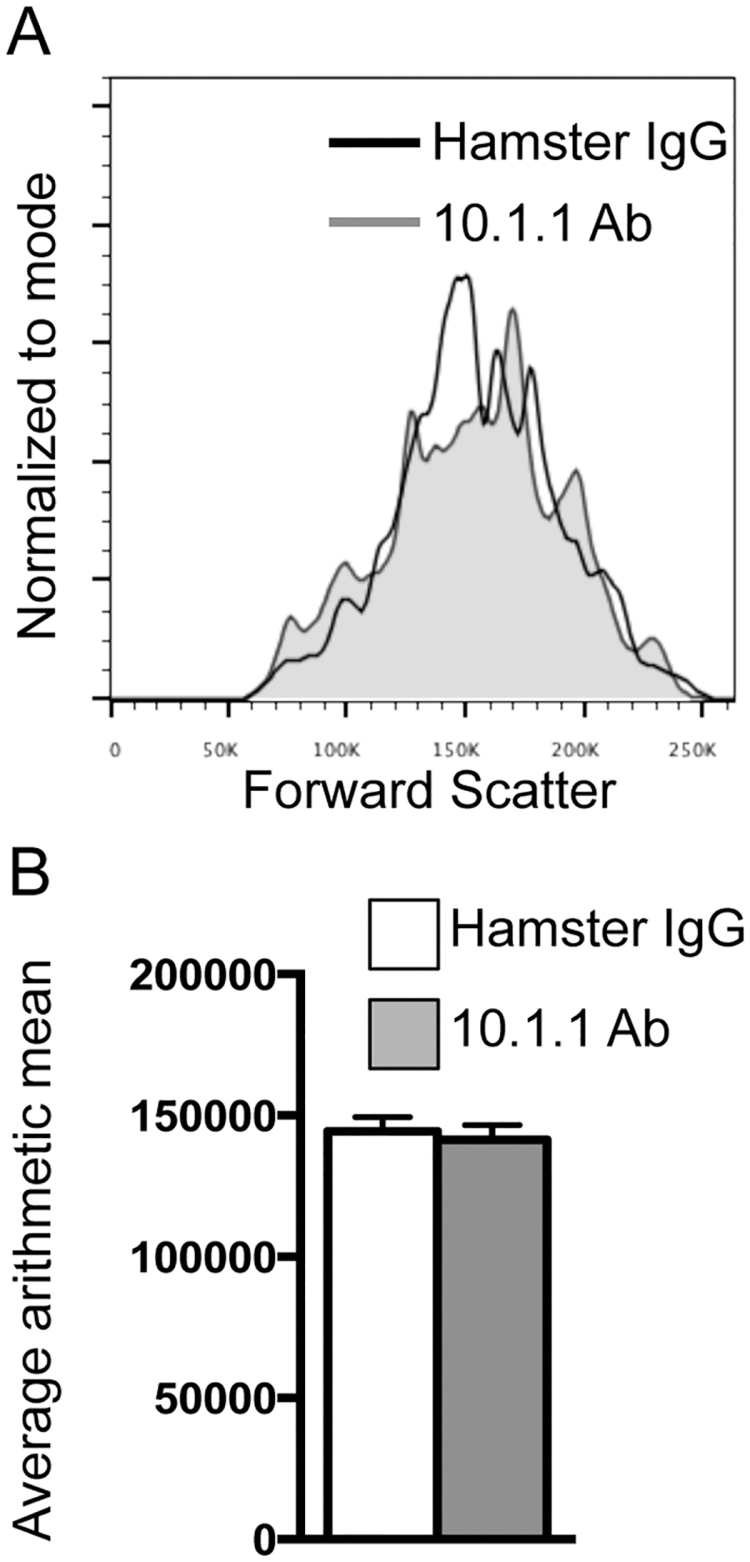
10.1.1 Ab-induced lymphangiogenesis is not due to enlargement of LECs. A). Pooled LNs were enzymatically digested and analyzed by flow cytometry. No difference in forward scatter profiles was identified between control Hamster IgG- and 10.1.1 Ab-injected mice, indicating no difference in cell size between the two treatment groups. Overlay of forward scatter profile histograms gated on viable CD45- CD31+ Podoplanin+ LECs from representative samples of Hamster IgG- and 10.1.1 Ab-injected mice are shown. B). Bar graph represents the average arithmetic mean and standard error from 2 independent experiments. n = 6.

In addition to an increase in LN LEC number, the 10.1.1 Ab-induced lymphatic sinus growth could also potentially involve enlargement of LECs. To test this hypothesis, LNs were digested with collagenase and dispase and analyzed by flow cytometry for cell size. Comparison of forward scatter profiles of LECs (CD45-CD31+Podoplanin+ cells) demonstrated the same cell size in Hamster IgG-injected and 10.1.1 Ab-injected mice (Fig 5A). Analysis of six mice from each group demonstrated no difference in the arithmetic mean of the forward scatter profile of LECs (Fig 5B). Additionally, analysis of LYVE-1 stained sections did not identify any change in LN LEC size or morphology *in situ* after 10.1.1 Ab treatment (S2B Fig). Although dilation of collecting lymphatic vessels has been identified [46], we did not observe any dilation or thinning of the LN lymphatic sinuses that could contribute to their rapid growth in 10.1.1. Ab-treated mice (Fig 3A).

**Fig 6:**
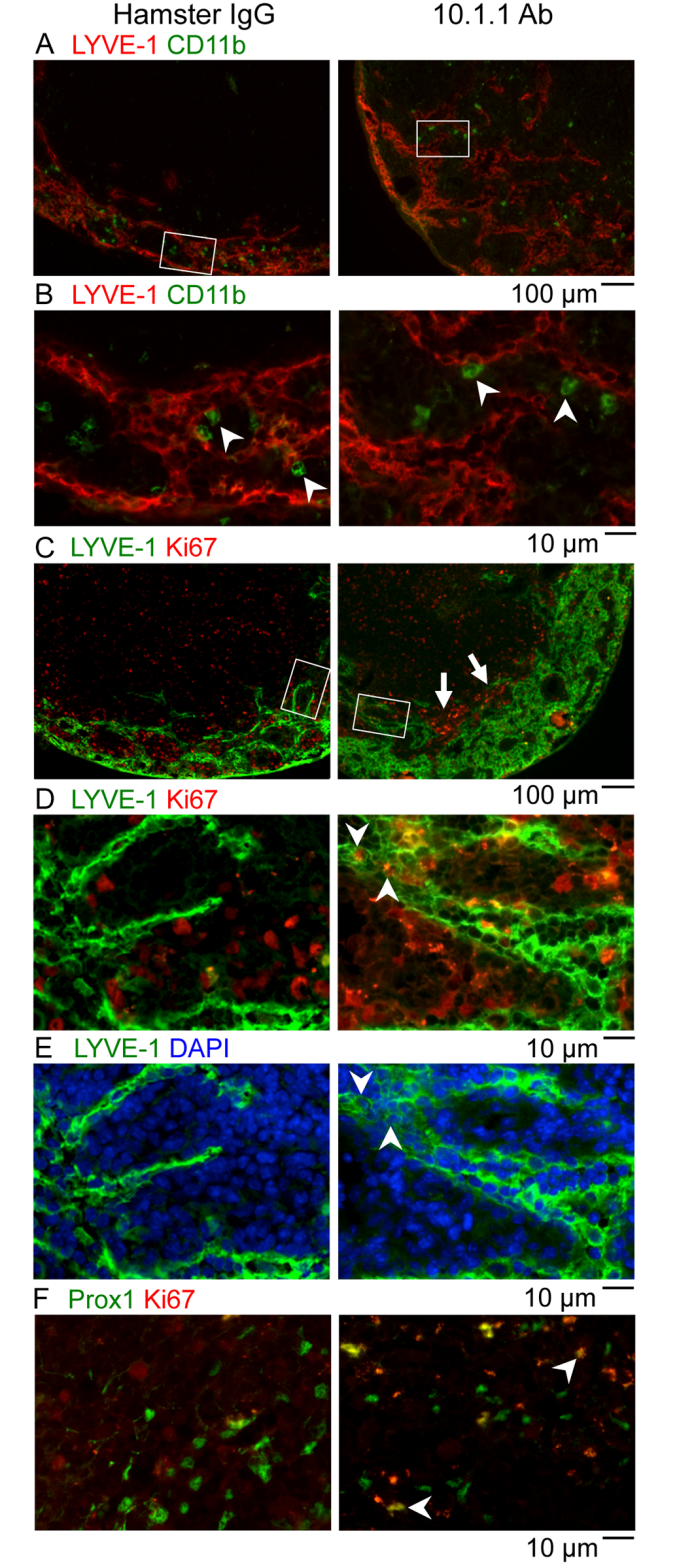
10.1.1 Ab induces proliferation of lymph node LECs and non-LECs. A-B). Popliteal LN sections of control Hamster IgG-injected or 10.1.1 Ab-injected mice were stained with anti-LYVE-1 antibodies (red), and anti-CD11b (green). White boxes on low magnification images (A) identify the region of medullary sinuses shown at high magnification in (B). No double-positive LYVE-1+ CD11b+ cells were identified in either treatment condition, indicating that CD11b+ cells do not directly contribute to lymphatic sinus growth. The representation or distribution of CD11b+ macrophages on each sinus (arrowheads) also did not change, indicating that sinus macrophages proportionally coat the growing lymphatic sinuses. C-D). Popliteal LN sections of control Hamster IgG-injected or 10.1.1 Ab-injected mice were stained with anti-LYVE-1 (green) and anti-Ki67 Abs (red) to identify proliferating LECs. White boxes on 10x images (C) identify the 40x regions of the medullary sinuses shown in D and E. A number of LYVE-1+ Ki67+ LECs (e.g. arrowheads) were identified near the growing edge of the medullary lymphatics in LNs from 10.1.1 Ab-injected mice but not control Hamster IgG-injected mice. Proliferating LYVE-1- Ki67+ non-LECs were clustered adjacent to the medullary lymphatic sinuses in LNs from 10.1.1 Ab-injected mice (C, arrows). E). The Ki67+ cells are DAPI-positive (e.g. compare arrowheads in D and E) confirming nuclear localization of the Ki67 immunostaining. F). Adjacent sections were stained with anti-Prox-1 (green) and anti-Ki67 (red). A number of nuclear Prox-1+ Ki67+ proliferating LECs (e.g. arrowheads) were identified in the same regions of the growing lymphatic sinuses in LNs from 10.1.1 Ab-injected but not hamster IgG-injected mice. Scale bars are indicated.

Macrophages can contribute to the growth of peripheral lymphatic vessels, for example by directly incorporating into lymphatic vessels in the inflamed diaphragm [25]. These incorporating macrophages express both CD11b and LYVE-1 markers. To determine whether the 10.1.1 Ab-induced LN sinus expansion involves incorporation of CD11b+ macrophages, popliteal LN sections were stained for LYVE-1 and CD11b expression. CD11b+ macrophages coated the LYVE-1+ lymphatic sinuses in both hamster IgG- and 10.1.1 Ab-injected mice. We were unable to detct any increase in macrophage representation or distribution on the growing sinuses in LNs from 10.1.1 Ab-treated mice (Fig 6A). Moreover, the staining of CD11b+ macrophages (Fig 6B, arrows) and LYVE-1+ LECs remained distinct, indicating that double-positive CD11b+ LYVE-1+ macrophages do not contribute to LN lymphatic sinus growth after 10.1.1 Ab treatment.

### 10.1.1 Ab induces LN LEC proliferation *in vivo*

We sought to determine whether 10.1.1 Ab-mediated LN lymphangiogenesis involves LEC proliferation similar to that identified *in vitro* (Fig 1). LEC proliferation was assessed *in situ* by immunofluorescent staining of popliteal LN sections for LEC LYVE-1 and the Ki67 proliferation marker. While proliferating cells are found throughout the LNs of hamster and 10.1.1 Ab-treated mice, clustered Ki67+ cells were prominent features near the edge of the medullary lymphatic sinuses in LNs from 10.1.1 Ab-injected mice (arrows, Fig 6C, right panel), suggesting that the 10.1.1 Ab also activates proliferation of non-LECs. Examination at higher magnification identified abundant Ki67+ LECs within LYVE-1+ lymphatic sinuses (e.g. arrowheads, Fig 6D right panel), in addition to Ki67+ non-LECs adjacent to the sinuses in LNs from 10.1.1 Ab-treated mice. The Ki67 nuclei colocalized with DAPI staining, confirming that the Ki67 Ab staining identifies nuclei of proliferating LECs (e.g. arrowheads, Fig 6E, right panel) as well as those of proliferating non-LECs. Immunostaining with the LEC-specific nuclear Prox1 Ab [43] and Ki67 Ab in similar areas of adjacent sections of the LNs identified very few Prox1+ Ki67+ cells in LNs from hamster IgG-treated mice (Fig 6E, left panel), while they were abundant in LNs from 10.1.1 Ab-injected mice (e.g. arrowheads, Fig 6E, right panel). These findings demonstrate that 10.1.1 Ab treatment increases proliferation of lymphatic sinus LECs as well as other cell types within LNs. However, spleens from 10.1.1 Ab-treated animals demonstrated no difference in the representation or localization of KI67+ cells in the red pulp where the 10.1.1 Ab also binds to splenic stromal cells, or in the white pulp (S2C Fig). These findings demonstrate a specific effect of the 10.1.1 Ab within LNs to increase LEC and non-LEC proliferation.

### Proliferating non-LECs accompany expansion of LN lymphatic sinuses but do not physically contribute to lymphatic sinus growth

**Fig 7:**
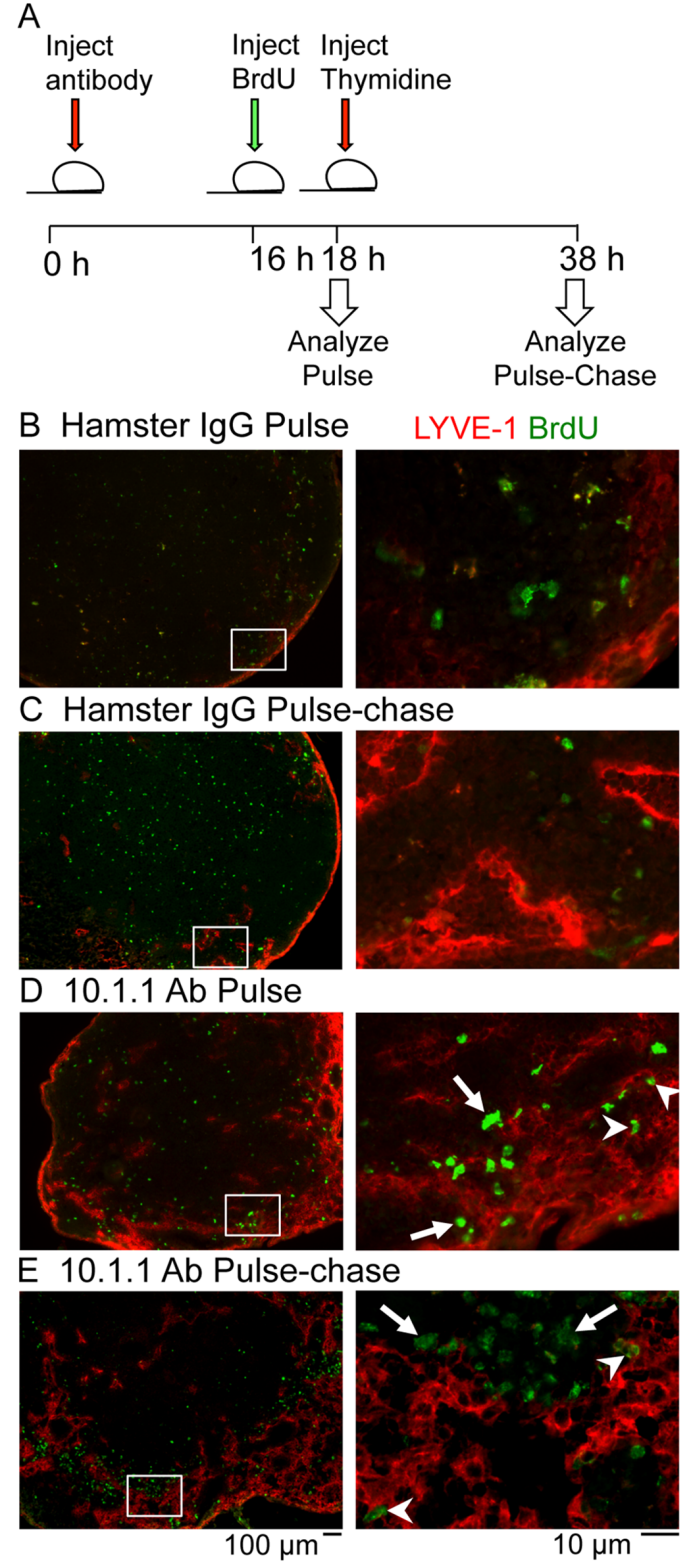
Lymph node lymphangiogenesis involves LEC and non-LEC proliferation. A). Schematic of the experimental timeline for the BrdU pulse-chase experiment. Mice were injected i.p. 10.1.1 Ab or control Hamster IgG at 0 h. At 16 h, mice were injected i.p. with BrdU. Two h after BrdU administration, mice were either sacrificed as part of the pulse cohort or injected i.p. with thymidine to stop further BrdU incorporation. The pulse-chase cohort was sacrificed at 38 h after Ab injection. B-E). Popliteal LN sections were stained with anti-LYVE-1 (red) and anti-BrdU (green) antibodies. White boxes on low magnification images (left panels) identify the medullary sinus regions shown at higher magnification in the right panels. LNs from Hamster IgG-injected mice from pulse (B) and pulse-chase (C) cohorts display similar LYVE-1 staining patterns and no BrdU labeling of LYVE-1+ cells. BrdU-positive LYVE-1+ LECs are identified in the pulse-labeled (D, arrowheads) and pulse chase-labeled LNs (E, arrowheads) from 10.1.1 Ab-injected mice. Most of the BrdU-positive LYVE-1- non-LEC remain clustered near the growing edge of the sinus in the pulse (D, arrows) and pulse-chase cohorts (E, arrows) indicating that these proliferating cells do not contribute directly to the sinus growth. Scale bars are indicated.

The appearance of proliferating non-LECs near the growing edge of the medullary sinuses in 10.1.1 Ab-injected mice led us to investigate whether these cells could directly contribute to rapid lymphatic sinus growth. Peripheral lymphatic vessel growth in response to inflammation can involve the incorporation of non-LEC cell types such as macrophages [25–27]. To test whether the proliferating non-LECs physically contribute to lymphatic sinus growth, a BrdU pulse-chase experiment was performed (Fig 7A). At 16 h after 10.1.1 or Hamster Ab injection, mice were injected with BrdU and the pulse cohort was harvested. For the pulse-chase cohort, thymidine chase was injected at 18 h and mice were then analyzed at 38 h after 10.1.1 Ab injection. As expected, control Hamster IgG-injected pulse-chase animals demonstrated no increase in lymphatic sinus area at 16 h (Fig 7B, left panel) or 38 h (Fig 7C, left panel) after Ab injection. Additionally, double-positive BrdU+LYVE-1+ cells were not identified in the pulse (Fig 7B, right panel) or pulse-chase cohorts in LNS from hamster IgG-injected mice (Fig 7C, right panel), demonstrating that there is not much basal proliferation of LN LECs. In contrast, at 16 h after 10.1.1 Ab injection BrdU-pulsed animals demonstrated increased lymphatic sinus area (Fig 7D, left panel), and clustering of BrdU-labeled cells within as well as adjacent to the growing medullary lymphatic sinuses (Fig 7D, right panel). The BrdU-labeled nuclei were identified as LYVE-1+ LECs (arrowheads, Fig 7D right panel), as well as LYVE-1- non-LECs located next to the sinuses (arrows, Fig 7D right panel).

The pulse-chase cohort of animals also demonstrated increased lymphatic sinuses at 38 h after 10.1.1 Ab injection (Fig 7E, left panel). The BrdU-labelled cells that had previously incorporated BrdU during the 16–18 h pulse were found in the medullary sinus LECs (e.g. arrowheads, Fig 7E, right panel,), as well as in non-LECs adjacent to the lymphatic sinuses (e.g. arrows, Fig 7E, right panel). Additional examples of BrdU incorporation again demonstrate incorporation of some of the BrdU-labeled cells into the lymphatic sinuses in LNs from 10.1.1 Ab-injected and pulse-chase-labeled mice (arrowheads, middle panel, S4B Fig), and persistence of BrdU-labeled non-LEC adjacent to sinuses (arrows, middle panel, S4B Fig). The co-localization of nuclear DAPI with BrdU is demonstrated in proliferating LECs (arrowheads, middle and right panels, S4B Fig) and in proliferating non-LECs (arrows, middle and right panels, S4B Fig). The pulse-labeled LECs thus contribute to LN lymphatic sinus growth, while proliferating non-LECs adjacent to the medullary lymphatic sinuses are not obviously incorporated into the growing lymphatic sinuses. Taken together, the pulse- and pulse-chase experiments demonstrate that the 10.1.1 Ab induces LN LEC proliferation, while also activating the proliferation of other LN cell types that may indirectly support rapid LN lymphangiogenesis without directly incorporating into the sinuses.

### 10.1.1 Ab treatment does not induce lymphatic vessel growth

In addition to expression on LN LECs, the 10.1.1 Ab binds to stromal epithelium in the red pulp of the spleen [30], and to the LECs of initial and collecting lymphatic vessels [12]. To investigate the impact of 10.1.1 Ab binding on other mCLCA1-expressing cells, the spleen and lymphatic vessels of the small intestine and dermis were examined. Immunostaining of the spleen identified no gross alteration in 10.1.1 Ab staining of the splenic red pulp, or in the distribution of Ki67+ proliferating cells (S2C Fig). DAPI nuclear staining also demonstrated no difference in architecture of the red (R) and white (W) pulp regions (S2D Fig). Macrophage staining with the F4/80 Ab [47] and splenic stromal epithelial staining with 10.1.1 Ab also did not identify any gross alterations in the red pulp structure or composition (S2E Fig).

The jejunum in the small intestine features lacteal initial lymphatic vessels within the villi and also mucosal collecting lymphatic vessels in the submucosa. The lacteals (arrows, S2F Fig) as well as the mucosal lymphatic vessels (arrowheads, S2F Fig) showed no difference in size or morphology 23 h after hamster IgG or 10.1.1 Ab treatment. Immunostaining for LYVE-1 and Ki67 did not identify LEC proliferation in the lacteals (arrows, S2G Fig) or in the mucosal lymphatic vessels (arrowheads, S2G Fig) from hamster IgG or 10.1.1 Ab-injected mice.

In the dermis, there was also no difference in the abundance or size of lymphatic vessels in 8.1.1- versus 10.1.1 Ab-treated mice at 23 h after 10.1.1 Ab injection (arrows, S2H Fig). These findings indicate that the immediate impact of the 10.1.1 Ab to stimulate LEC proliferation and tissue remodeling within the first 23 h is restricted to the LN lymphatic sinuses, despite expression of the mCLCA1 antigen on splenic red pulp stroma and on lymphatic vessels.

## Discussion

While LN lymphangiogenesis is a feature of the immune response that is involved in multiple disease states, the mechanisms by which lymphocytes and leukocytes promote lymphatic sinus growth are not known. Studies of peripheral lymphatic vessel growth have identified proliferation and migration [24] as major contributors, as well as incorporation of bone marrow-derived CD11b+ macrophages [25–27]. We did not identify accelerated incorporation of CD11b+ macrophages into lymphatic sinuses after 10.1.1 Ab treatment, suggesting that this type of incorporation could be limited to peripheral lymphatic vessels. However, 10.1.1 Ab treatment did specifically induce LEC proliferation both *in vitro* and *in vivo* within LNs. Basal LN LEC proliferation was difficult to detect in control LNs, suggesting that activation of proliferation makes a major contribution to the rapid induction of LN lymphangiogenesis by 10.1.1 Ab.

The mechanism by which 10.1.1 Ab binding to mCLCA1 induces LN LEC proliferation remains to be determined. Little is known yet about mCLCA1 function, as despite its name this protein does not appear to function as a calcium-activated chloride channel [32], while a proposed hydrolase activity has not been tested [33]. The 10.1.1 Ab induction of lymphatic sinus growth appears to be a specific consequence of binding to LN LEC mCLCA1 rather than by effects on some other target, due to the rapidity of the lymphangiogenesis induced within less than a day, versus the weeks to months required to induce lymphatic sinus growth during immune responses [5, 8] or tumor formation [15, 16]. Moreover, the 10.1.1 Ab binding is specific for LECs and not other LN stromal cell types [2, 12]. The ability of the 10.1.1 Ab to induce LN lymphangiogenesis appears to involve specific mCLCA1 binding, as the 8.1.1 Ab that binds to LN LEC podoplanin has no effect on the lymphatic sinuses. Finally, the 10.1.1, 8.1.1, and hamster Abs contained similar endotoxin levels and were simultaneously purified in several batches in the same antibody production facility, so that it is unlikely that some non-specific component of the 10.1.1 Ab preparation mediates these effects. Taken together, these findings suggest that the 10.1.1 Ab rapidly and directly induces LN LEC proliferation and sinus growth by binding to mCLCA1 on LN LECs. It remains to be determined whether the rapid 10.1.1 Ab induction of LN lymphangiogenesis is ed by secondary mechanisms in vivo, such as induction of stromal or immune cell growth factor or cytokine production [7, 23, 48].

Our previous finding that mCLCA1 strongly binds to LFA1 to mediate LEC-lymphocyte adhesion [30], and that LFA1-expressing B cells promote LN lymphatic sinus growth [5, 16] suggest the hypothesis that the 10.1.1 Ab acts as an LFA1 agonist to stimulate lymphangiogenesis. We found that both 10.1.1 Ab and lymphocytes strongly blocked tube formation *in vitro*, consistent with the idea that the 10.1.1 Ab mimics lymphocyte LFA-1 interactions with mCLCA1 to influence LEC adhesion and migration. We were surprised to find that the 10.1.1 Ab and lymphocytes blocked rather than stimulated tube formation *in vitro*, in contrast to their strong effects to promote LN lymphangiogenesis *in vivo*. Further studies will be required to distinguish whether these *in vivo* treatments activate LEC migration in a manner that blocks tube formation, or if they prevent mCLCA1 adhesion activity required for stable tube formation *in vitro*. mCLCA1 also binds to Mac1 [30], so that the effects of 10.1.1 Ab *in vivo* alternatively could involve monocytes or dendritic cells, which can also promote LN lymphatic sinus growth during inflammation [22]. Much remains to be learned about these lymphatic/immune interactions and the involvement of mCLCA1 in regulation of LN lymphangiogenesis. In addition, proof that the 10.1.1 Ab induces LN lymphangiogenesis via mCLCA1-integrin interactions will require the generation and analysis of LN lymphatic sinus growth in mCLCA1-, LFA1-, and Mac1-null mice.

The effect of the 10.1.1 Ab to induce lymphangiogenesis was restricted to the medullary lymphatic sinuses of the LN, despite expression of the mCLCA1 protein by all LN lymphatic sinus endothelium and by lymphatic vessels, indicating a distinct role for this protein in LN medullary sinuses. It is possible that a second factor restricted to the medullary sinuses cooperates with mCLCA1 to regulate LN lymphangiogenesis. Alternatively, the timing of lymphangiogenesis could be tissue-dependent. The medullary lymphatic sinuses may be poised to undergo rapid growth within a day, while the cortical lymphatic sinuses or peripheral lymphatic vessels could respond more slowly.

While 10.1.1 Ab binding induces proliferation of LN LECs, the expansion of additional cell types accompanies 10.1.1 Ab-induced LN remodeling *in vivo*. The periodic CD11b+ macrophage coating of the lymphatic sinuses is preserved during sinus growth, indicating that macrophages proportionately increase along with the sinuses. However, these macrophages do not become LYVE-1-positive, so that they cannot account for the rapid increase in LYVE1-+ lymphatic sinus area. 10.1.1 Ab also proportionately increased the other CD45- LN stromal subpopulations including BEC, FRC, and DN stroma. This coordinate expansion of all of the LN stromal populations *in vivo* also occurs during LN remodeling in inflammation models of LN lymphangiogenesis [66]. Our findings suggest that the expansion of these stromal cell populations is likely secondary to the 10.1.1 Ab binding to LN LECs. The 10.1.1 Ab-treated mice also displayed clustered proliferating non-LECs surrounding the growing medullary lymphatic sinuses, which are negative for LYVE-1, 10.1.1, Prox1, and CD11b (data not shown). While we have demonstrated that proliferating non-LECs generally do not incorporate into the lymphatic sinuses and remain LYVE-1-negative, these cells could potentially contribute molecular signals driving the expansion of stromal BEC, FRC, and DN stromal cells in 10.1.1 Ab-treated mice

LN lymphangiogenesis and remodeling has been shown to be induced by both innate and adaptive immune cells, and has been suggested to play an important role in the productive development of an immune response and in reinforcement of peripheral tolerance [9, 13, 49]. Our finding that the 10.1.1 Ab rapidly induces these LN alterations suggests that LEC mCLCA1 could exert a key regulatory role to initiate the complex program of LN remodeling during immune responses. While multiple factors regulating peripheral lymphatic vessel growth have been identified, much less is known about regulation of LN lymphatic sinus growth, so that further investigation of mCLCA1 involvement could give insight to the mechanism by which B lymphocytes specifically induce growth of LN lymphatic sinuses. LN lymphangiogenesis promotes tumor metastasis [14] and may regulate the severity of arthritis flares [18, 50], so that an underanding of the cells and molecules regulating LN lymphatic sinus growth could lead to new strategies for therapeutic manipulation of immune responses. Further investigation will determine whether and how mCLCA1 and its binding partners LFA1 and Mac1 integrins could initiate normal or abnormal LN lymphangiogenesis in immune responses or disease.

## Supporting Information

S1 Fig 10.1.1 Ab induces medullary lymphatic sinus growth. A). Popliteal LN demonstrates lymphatic sinus growth in the medulla at 23 h after 10.1.1 Ab-injection. B). Nuclear DAPI staining of the section in (A) identifies the cortical region containing primary B cell follicles (dashed line), which does not exhibit lymphangiogenesis.
(TIF)

S2 Fig 10.1.1 Ab induces rapid lymph node lymphangiogenesis but does not induce splenic alterations or expansion of lymphatic vessels. A). Axillary LN sections were stained with anti-LYVE-1 Ab (green) to identify lymphatic endothelium, and nuclei were stained with DAPI (blue). Increased lymphatic sinuses were identified in 10.1.1 Ab-injected mice (right panel) compared to Hamster IgG injected controls (left panel). Three mice per treatment were analyzed. B). LYVE-1 immunostaining of lymphatic sinuses shown at higher magnification demonstrates that the lymphatic sinuses mainly contain LYVE-1+ cells, and that the diameter of the sinuses is similar in Hamster IgG- and 10.1.1 Ab-injected mice. C). Spleen cryosections from Hamster IgG- and 10.1.1 Ab-injected mice were immunostained with 10.1.1 Ab (red) to identify the stromal cells of the red pulp and with anti-Ki67 Ab (green) to identify proliferating cells. No alteration in the architecture of the red (R) or white (W) pulp was observed in 10.1.1 Ab-treated mice. No change in number or distribution of Ki67+ proliferating cells was observed in response to 10.1.1 Ab treatment. Six mice per treatment were analyzed. D). DAPI nuclear staining demonstrates no gross differences in the architecture of the red and white pulp of spleens from Hamster IgG or 10.1.1 Ab-injected mice. E). 10.1.1 Ab (red) and F4/80 macrophage (green) staining demonstrates similar morphology of splenic stromal epithelial and marocophage populations in spleens, respectively. F). Sections of the small intestine (jejunum) were stained with anti-LYVE-1 antibody (red) to identify lymphatics and counterstained with nuclear DAPI (blue). The abundance and morphology of lacteal lymphatic vessels (arrows) or mucosal lymphatic vessels (arrowheads) was similar in jejunum from Hamster IgG and 10.1.1 Ab-treated mice. Two mice per treatment were analyzed. G). Anti-LYVE-1 (red) and anti-Ki67 (green) staining shows that there are occasional proliferating cells in jejunum, while proliferating Ki67-positive LECs are not identified in the lacteals (arrows) or mucosal lymphatic vessels (arrowheads) in hamster IgG- or 10.1.1 Ab-injected mice. Two mice per treatment were analyzed. H). Sections of skin from the feet of mice injected with 8.1.1 Ab or 10.1.1 Ab were stained with anti-LYVE-1, using HRP immunohistochemical detection with Vector VIP. The abundance and morphology of purple dermal initial lymphatic vessels (arrows) was similar in hamster IgG or 10.1.1 Ab-injected mice. Four mice per treatment were analyzed. Scale bars are indicated.
(TIF)

S3 Fig Flow cytometry of stroma identifies LN stromal subsets. A). Viable cells from LN stromal digests were selected using forward (FSC) and side scatter (SSC) gating as indicated. There was no difference in viability between hamster IgG- or 10.1.1 Ab-injected populations (n = 6). B). CD45- cells were separated into the four stromal subsets (FRC, LEC, DN, BEC) using Podoplanin and CD31 antibodies.
(TIF)

S4 Fig BrdU antibody labels nuclei of proliferating cells within LNs. A). A second example of an LN from a pulse-labeled 10.1.1 Ab-injected mouse was immunostained with anti-LYVE-1 (red) and with anti-BrdU (green) antibodies. The LYVE-1 and BrdU-stained region outlined by the white box is shown at higher magnification in the middle panel, while the right panel shows higher magnification of the same section immunostained with LYVE-1 in combination with blue DAPI staining of nuclei. BrdU immunostaining colocalizes with DAPI nuclear staining (arrows). B). A second example of an LN from a pulse-chase-labeled mouse immunostained for LYVE-1 and BrdU. The white boxed area is shown at higher magnification in the middle panel, demonstrating increased proliferation of LECs (e.g. arrowheads) and non-LECs (e.g. arrows). The right panel shows LYVE-1 staining in combination with DAPI staining of nuclei. BrdU immunostaining colocalizes with DAPI nuclear staining in LYVE-1- non-LECs (arrows), and in LYVE-1+ LECs (arrowheads). Scale bars are indicated.
(TIF)

## References

[1] Card CM, Yu SS, Swartz MA. Emerging roles of lymphatic endothelium in regulating adaptive immunity. Journal of Clinical Investigation. 2014;124: 943–52. 10.1172/JCI73316 24590280 PMC3938271

[2] Cohen JN, Guidi CJ, Tewalt EF, Qiao H, Rouhani SJ, Ruddell A, et al Lymph node-resident lymphatic endothelial cells mediate peripheral tolerance via Aire-independent direct antigen presentation. J Exp Med. 2010;207: 681–8. 10.1084/jem.20092465 20308365 PMC2856027

[3] Fletcher AL, Lukacs-Kornek V, Reynoso ED, Pinner SE, Bellemare-Pelletier A, Curry MS, et al Lymph node fibroblastic reticular cells directly present peripheral tissue antigen under steady-state and inflammatory conditions. J Exp Med. 2010;207: 689–97. 10.1084/jem.20092642 20308362 PMC2856033

[4] Lund AW, Duraes FV, Hirosue S, Raghavan VR, Nembrini C, Thomas SN, et al VEGF-C Promotes Immune Tolerance in B16 Melanomas and Cross-Presentation of Tumor Antigen by Lymph Node Lymphatics. Cell Rep. 2012;1: 191–9. 10.1016/j.celrep.2012.01.005 22832193

[5] Angeli V, Ginhoux F, Llodra J, Quemeneur L, Frenette PS, Skobe M, et al B cell-driven lymphangiogenesis in inflamed lymph nodes enhances dendritic cell mobilization. Immunity. 2006;24: 203–15. 16473832 10.1016/j.immuni.2006.01.003

[6] Chyou S, Benahmed F, Chen J, Kumar V, Tian S, Lipp M, et al Coordinated regulation of lymph node vascular-stromal growth first by CD11c+ cells and then by T and B cells. J Immunol. 2011;187:5558–67. 10.4049/jimmunol.1101724 22031764 PMC3221869

[7] Kumar V, Scandella E, Danuser R, Onder L, Nitschke M, Fukui Y, et al Global lymphoid tissue remodeling during a viral infection is orchestrated by a B cell-lymphotoxin-dependent pathway. Blood. 2010;115: 4725–33. 10.1182/blood-2009-10-250118 20185585

[8] Liao S, Ruddle NH. Synchrony of high endothelial venules and lymphatic vessels revealed by immunization. J Immunol. 2006;177: 3369–79. 16920978 10.4049/jimmunol.177.5.3369

[9] Benahmed F, Ely S, Lu TT. Lymph node vascular-stromal growth and function as a potential target for controlling immunity. Clin Immunol. 2012;144: 109–16. 10.1016/j.clim.2012.05.004 22717771 PMC3932544

[10] Chang JE, Turley SJ. Stromal infrastructure of the lymph node and coordination of immunity. Trends Immunol. 2015;36: 30–9. 10.1016/j.it.2014.11.003 25499856

[11] Ji RC. Lymph node lymphangiogenesis: a new concept for modulating tumor metastasis and inflammatory process. Histol Histopathol. 2009;24: 377–84. 19130407 10.14670/HH-24.377

[12] Ruddell A, Mezquita P, Brandvold KA, Farr A, Iritani BM. B lymphocyte-specific c-Myc expression stimulates early and functional expansion of the vasculature and lymphatics during lymphomagenesis. American Journal of Pathology. 2003;163: 2233–45. 14633598 10.1016/S0002-9440(10)63581-X PMC1892400

[13] Swartz MA. Immunomodulatory roles of lymphatic vessels in cancer progression. Cancer immunology research. 2014;2: 701–7. 10.1158/2326-6066.CIR-14-0115 25092811

[14] Ruddell A, Harrell MI, Furuya M, Kirschbaum SB, Iritani BM. B lymphocytes promote lymphogenous metastasis of lymphoma and melanoma. Neoplasia. 2011;13: 748–57. 21847366 10.1593/neo.11756 PMC3156665

[15] Ruddell A, Kelly-Spratt KS, Furuya M, Parghi SS, Kemp CJ. p19/Arf and p53 suppress sentinel lymph node lymphangiogenesis and carcinoma metastasis. Oncogene. 2008;27: 3145–55. 18059331 10.1038/sj.onc.1210973

[16] Harrell MI, Iritani BM, Ruddell A. Tumor-induced sentinel lymph node lymphangiogenesis and increased lymph flow precede melanoma metastasis. American Journal of Pathology. 2007;170: 774–86. 17255343 10.2353/ajpath.2007.060761 PMC1851877

[17] Tan KW, Yeo KP, Wong FH, Lim HY, Khoo KL, Abastado JP, et al Expansion of cortical and medullary sinuses restrains lymph node hypertrophy during prolonged inflammation. J Immunol. 2012;188: 4065–80. 10.4049/jimmunol.1101854 22430738

[18] Guo R, Zhou Q, Proulx ST, Wood R, Ji RC, Ritchlin CT, et al Inhibition of lymphangiogenesis and lymphatic drainage via vascular endothelial growth factor receptor 3 blockade increases the severity of inflammation in a mouse model of chronic inflammatory arthritis. Arthritis Rheum. 2009;60: 2666–76. 10.1002/art.24764 19714652 PMC2810533

[19] Li J, Kuzin I, Moshkani S, Proulx ST, Xing L, Skrombolas D, et al Expanded CD23(+)/CD21(hi) B cells in inflamed lymph nodes are associated with the onset of inflammatory-erosive arthritis in TNF-transgenic mice and are targets of anti-CD20 therapy. J Immunol. 2010;184: 6142–50. 10.4049/jimmunol.0903489 20435928 PMC2874087

[20] Webster B, Ekland EH, Agle LM, Chyou S, Ruggieri R, Lu TT. Regulation of lymph node vascular growth by dendritic cells. J Exp Med. 2006;203: 1903–13. 16831898 10.1084/jem.20052272 PMC2118366

[21] Halin C, Tobler N, Vigl B, Brown L, Detmar M, Brakenhielm E, et al VEGF-A produced by chronically inflamed tissue induces lymphangiogenesis in draining lymph nodes. Blood. 2007;110: 31258–3167. 10.1182/blood-2007-01-066811 PMC2200913 17625067

[22] Benahmed F, Chyou S, Dasoveanu D, Chen J, Kumar V, Iwakura Y, et al Multiple CD11c+ cells collaboratively express IL-1beta to modulate stromal vascular endothelial growth factor and lymph node vascular-stromal growth. J Immunol. 2014;192: 4153–63. 10.4049/jimmunol.1301765 24659690 PMC4207367

[23] Onder L, Narang P, Scandella E, Chai Q, Iolyeva M, Hoorweg K, et al IL-7-producing stromal cells are critical for lymph node remodeling. Blood. 2012;120: 4675–83. 10.1182/blood-2012-03-416859 22955921 PMC3952724

[24] Karpanen T, Alitalo K. Molecular biology and pathology of lymphangiogenesis. Annu Rev Pathol. 2008;3: 367–97. 18039141 10.1146/annurev.pathmechdis.3.121806.151515

[25] Hall KL, Volk-Draper LD, Flister MJ, Ran S. New model of macrophage acquisition of the lymphatic endothelial phenotype. PLoS One. 2012;7:e31794 10.1371/journal.pone.0031794 22396739 PMC3292559

[26] Maruyama K, Ii M, Cursiefen C, Jackson DG, Keino H, Tomita M, et al Inflammation-induced lymphangiogenesis in the cornea arises from CD11b-positive macrophages. J Clin Invest. 2005;115: 2363–72. 16138190 10.1172/JCI23874 PMC1193872

[27] Zumsteg A, Baeriswyl V, Imaizumi N, Schwendener R, Ruegg C, Christofori G. Myeloid cells contribute to tumor lymphangiogenesis. PLoS One. 2009;4: e7067 10.1371/journal.pone.0007067 19759906 PMC2738969

[28] Lee JY, Park C, Cho YP, Lee E, Kim H, Kim P, et al Podoplanin-expressing cells derived from bone marrow play a crucial role in postnatal lymphatic neovascularization. Circulation. 2010;122: 1413–25. 10.1161/CIRCULATIONAHA.110.941468 20855662 PMC2989430

[29] Religa P, Cao R, Bjorndahl M, Zhou Z, Zhu Z, Cao Y. Presence of bone marrow-derived circulating progenitor endothelial cells in the newly formed lymphatic vessels. Blood. 2005;106: 4184–90. 16141354 10.1182/blood-2005-01-0226

[30] Furuya M, Kirschbaum SB, Paulovich A, Pauli BU, Zhang H, Alexander JS, et al Lymphatic endothelial murine chloride channel calcium-activated 1 is a ligand for leukocyte LFA-1 and Mac-1. J Immunol. 2010;185: 5769–77. 10.4049/jimmunol.1002226 20937843 PMC3367505

[31] Gandhi R, Elble RC, Gruber AD, Schreur KD, Ji HL, Fuller CM, et al Molecular and functional characterization of a calcium-sensitive chloride channel from mouse lung. J Biol Chem. 1998;273: 32096–101. 9822685 10.1074/jbc.273.48.32096

[32] Loewen ME, Forsyth GW. Structure and function of CLCA proteins. Physiol Rev. 2005;85: 1061–92. 15987802 10.1152/physrev.00016.2004

[33] Pawlowski K, Lepisto M, Meinander N, Sivars U, Varga M, Wieslander E. Novel conserved hydrolase domain in the CLCA family of alleged calcium-activated chloride channels. Proteins. 2006;63: 424–39. 16470849 10.1002/prot.20887

[34] Johnson LA, Prevo R, Clasper S, Jackson DG. Inflammation-induced uptake and degradation of the lymphatic endothelial hyaluronan receptor LYVE-1. J Biol Chem. 2007;282: 33671–80. 17884820 10.1074/jbc.M702889200

[35] Ruddell A, Croft A, Kelly-Spratt K, Furuya M, Kemp CJ. Tumors induce coordinate growth of artery, vein, and lymphatic vessel triads. BMC Cancer. 2014;14:354 10.1186/1471-2407-14-354 24886322 PMC4045915

[36] Springer TA. Traffic signals on endothelium for lymphocyte recirculation and leukocyte emigration. Annu Rev Physiol. 1995;57: 827–72. 7778885 10.1146/annurev.ph.57.030195.004143

[37] Farr A, Nelson A, Hosier S, Kim A. A novel cytokine-responsive cell surface glycoprotein defines a subset of medullary thymic epithelium in situ. J Immunology. 1993;150: 1160–71. 8432973

[38] Farr A, Nelson A, Hosier S. Characterization of an antigenic determinant preferentially expressed by type I epithelial cells in the murine thymus. Journal of Histochemistry & Cytochemistry. 1992;40: 651–64. 1374092 10.1177/40.5.1374092

[39] Jordan-Williams KL, Ruddell A. Culturing Purifies Murine Lymph Node Lymphatic Endothelium. Lymphat Res Biol. 2014;12: 144–9. 10.1089/lrb.2013.0053 24837301 PMC4171392

[40] Fletcher AL, Malhotra D, Acton SE, Lukacs-Kornek V, Bellemare-Pelletier A, Curry M, et al Reproducible isolation of lymph node stromal cells reveals site-dependent differences in fibroblastic reticular cells. Front Immunol. 2011;2: 35 10.3389/fimmu.2011.00035 22566825 PMC3342056

[41] Link A, Vogt TK, Favre S, Britschgi MR, Acha-Orbea H, Hinz B, et al Fibroblastic reticular cells in lymph nodes regulate the homeostasis of naive T cells. 2007;8: 1255–65. 10.1038/ni1513 17893676

[42] Ando T, Jordan P J T, Wang Y, Jennings MH, Houghton J, Alexander JS. Isolation and characterization of a novel mouse lymphatic endothelial cell line: SV-LEC. Lymphat Res Biol. 2005;3: 105–15. 16190815 10.1089/lrb.2005.3.105

[43] Hong YK, Harvey N, Noh YH, Schacht V, Hirakawa S, Detmar M, et al Prox1 is a master control gene in the program specifying lymphatic endothelial cell fate. Developmental Dynamics. 2002;225: 351–7. 12412020 10.1002/dvdy.10163

[44] Prevo R, Banerji S, Ferguson DJ, Clasper S, Jackson DG. Mouse LYVE-1 is an endocytic receptor for hyaluronan in lymphatic endothelium. J Biol Chem. 2001;276: 19420–30. 11278811 10.1074/jbc.M011004200

[45] Schacht V, Dadras SS, Johnson LA, Jackson DG, Hong YK, Detmar M. Up-regulation of the lymphatic marker podoplanin, a mucin-type transmembrane glycoprotein, in human squamous cell carcinomas and germ cell tumors. Am J Pathol. 2005;166: 913–21. 15743802 10.1016/S0002-9440(10)62311-5 PMC1602360

[46] Karnezis T, Shayan R, Caesar C, Roufail S, Harris NC, Ardipradja K, et al VEGF-D promotes tumor metastasis by regulating prostaglandins produced by the collecting lymphatic endothelium. Cancer Cell. 2012;21: 181–95. 10.1016/j.ccr.2011.12.026 22340592

[47] Austyn JM, Gordon S. F4/80, a monoclonal antibody directed specifically against the mouse macrophage. Eur J Immunol. 1981;11: 805–15. 7308288 10.1002/eji.1830111013

[48] Lukacs-Kornek V, Malhotra D, Fletcher AL, Acton SE, Elpek KG, Tayalia P, et al Regulated release of nitric oxide by nonhematopoietic stroma controls expansion of the activated T cell pool in lymph nodes. Nat Immunol. 2011;12: 1096–104. 10.1038/ni.2112 21926986 PMC3457791

[49] Tewalt EF, Cohen JN, Rouhani SJ, Engelhard VH. Lymphatic endothelial cells—key players in regulation of tolerance and immunity. Front Immunol. 2012;3: 305 10.3389/fimmu.2012.00305 23060883 PMC3460259

[50] Bouta EM, Li J, Ju Y, Brown EB, Ritchlin CT, Xing L, et al The role of the lymphatic system in inflammatory-erosive arthritis. Seminars in cell & developmental biology. 2015;38: 90–7. 25598390 10.1016/j.semcdb.2015.01.001 PMC4397133

