# Supplementary figures and images for: The Lymphatic Endothelial mCLCA1 Antibody Induces Proliferation and Growth of Lymph Node Lymphatic Sinuses
Source: PLoS One. 2016 May 25;11(5):e0156079. doi: 10.1371/journal.pone.0156079 (PMC4880189; doi:10.1371/journal.pone.0156079)

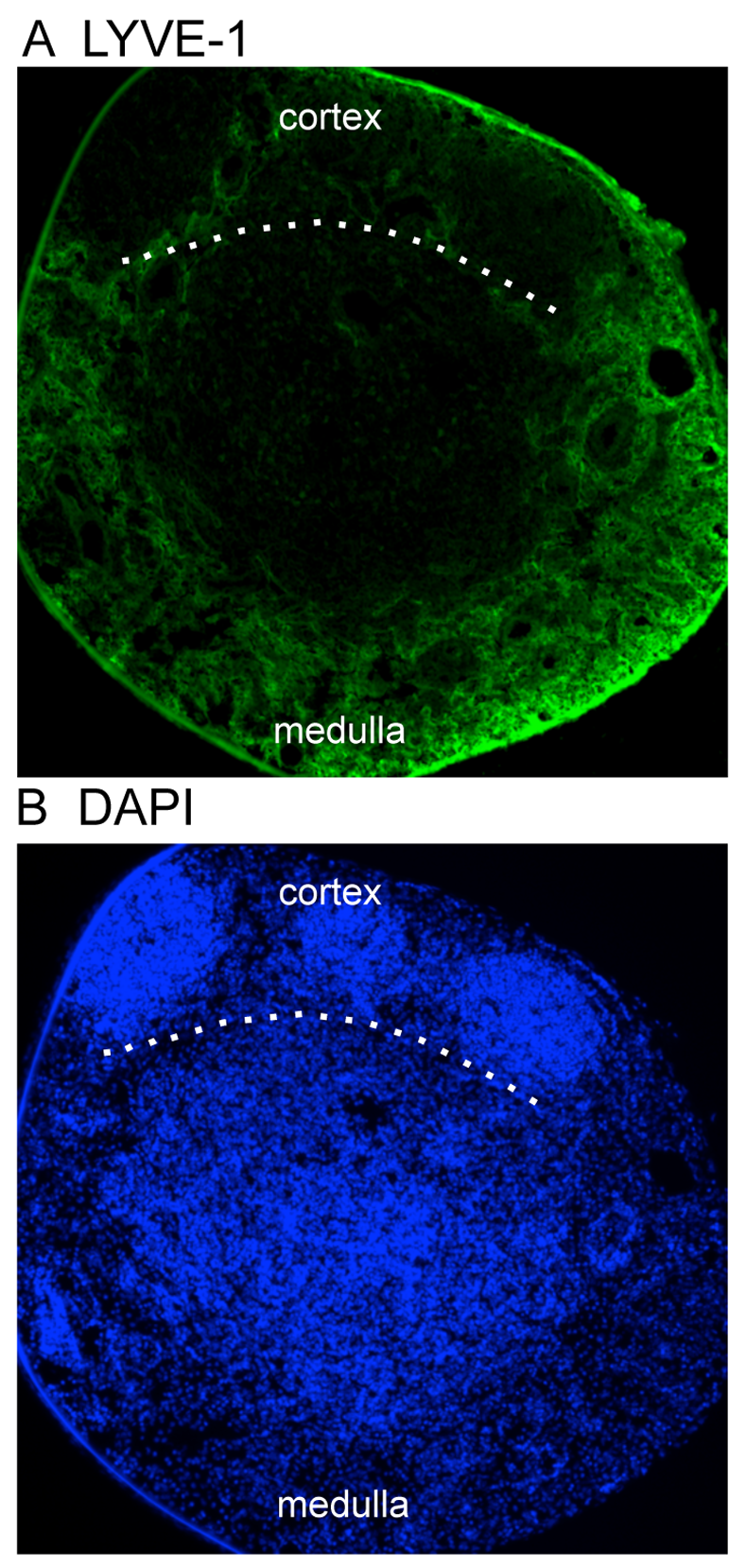

Supplement: S1 Fig — A). Popliteal LN demonstrates lymphatic sinus growth in the medulla at 23 h after 10.1.1 Ab-injection. B). Nuclear DAPI staining of the section in (A) identifies the cortical region containing primary B cell follicles (dashed line), which does not exhibit lymphangiogenesis. (TIF) [file pone.0156079.s001.tif]

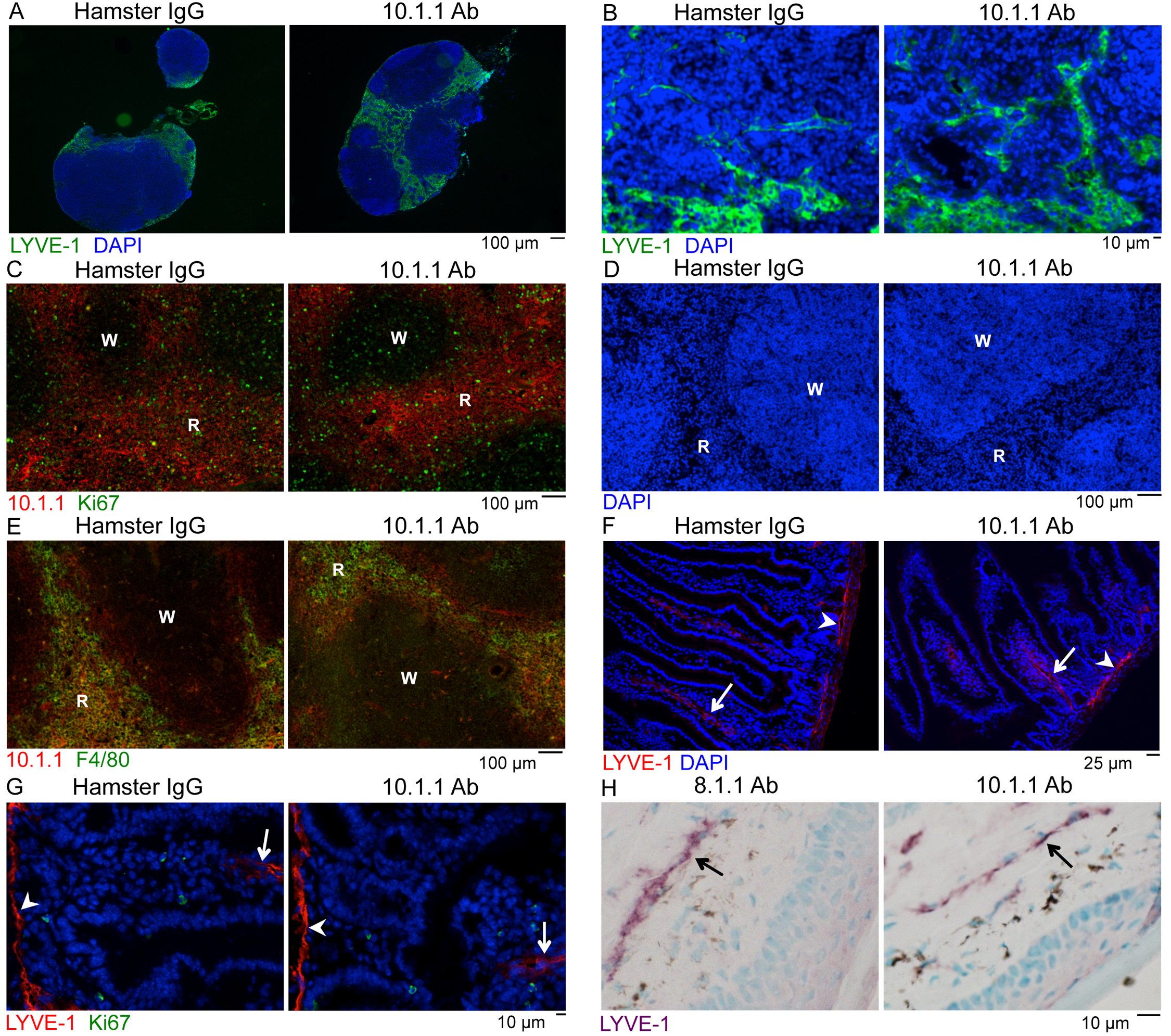

Supplement: S2 Fig — A). Axillary LN sections were stained with anti-LYVE-1 Ab (green) to identify lymphatic endothelium, and nuclei were stained with DAPI (blue). Increased lymphatic sinuses were identified in 10.1.1 Ab-injected mice (right panel) compared to Hamster IgG injected controls (left panel). Three mice per treatment were analyzed. B). LYVE-1 immunostaining of lymphatic sinuses shown at higher magnification demonstrates that the lymphatic sinuses mainly contain LYVE-1+ cells, and that the diameter of the sinuses is similar in Hamster IgG- and 10.1.1 Ab-injected mice. C). Spleen cryosections from Hamster IgG- and 10.1.1 Ab-injected mice were immunostained with 10.1.1 Ab (red) to identify the stromal cells of the red pulp and with anti-Ki67 Ab (green) to identify proliferating cells. No alteration in the architecture of the red (R) or white (W) pulp was observed in 10.1.1 Ab-treated mice. No change in number or distribution of Ki67+ proliferating cells was observed in response to 10.1.1 Ab treatment. Six mice per treatment were analyzed. D). DAPI nuclear staining demonstrates no gross differences in the architecture of the red and white pulp of spleens from Hamster IgG or 10.1.1 Ab-injected mice. E). 10.1.1 Ab (red) and F4/80 macrophage (green) staining demonstrates similar morphology of splenic stromal epithelial and marocophage populations in spleens, respectively. F). Sections of the small intestine (jejunum) were stained with anti-LYVE-1 antibody (red) to identify lymphatics and counterstained with nuclear DAPI (blue). The abundance and morphology of lacteal lymphatic vessels (arrows) or mucosal lymphatic vessels (arrowheads) was similar in jejunum from Hamster IgG and 10.1.1 Ab-treated mice. Two mice per treatment were analyzed. G). Anti-LYVE-1 (red) and anti-Ki67 (green) staining shows that there are occasional proliferating cells in jejunum, while proliferating Ki67-positive LECs are not identified in the lacteals (arrows) or mucosal lymphatic vessels (arrowheads) in ham [file pone.0156079.s002.tif]

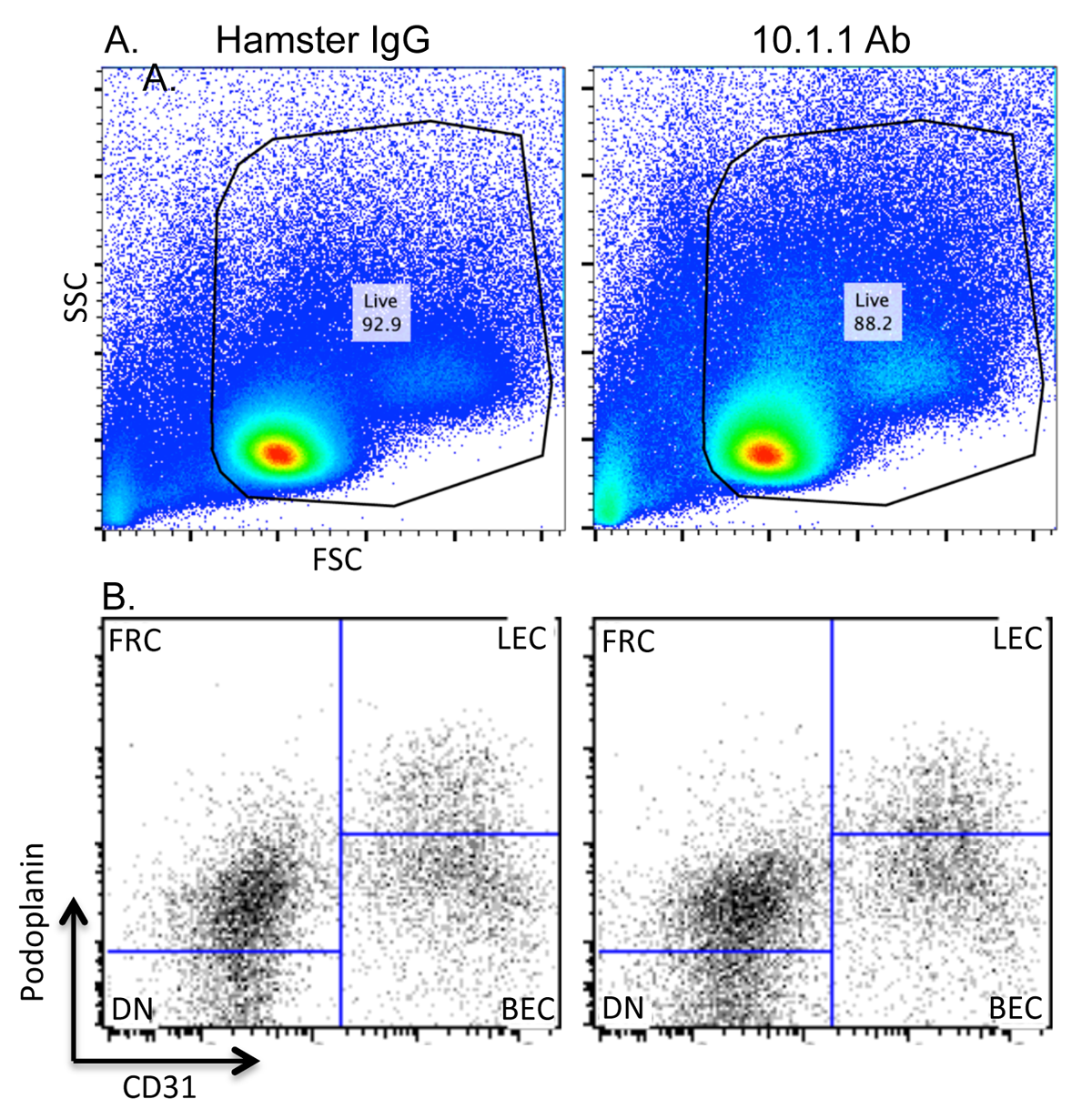

Supplement: S3 Fig — A). Viable cells from LN stromal digests were selected using forward (FSC) and side scatter (SSC) gating as indicated. There was no difference in viability between hamster IgG- or 10.1.1 Ab-injected populations (n = 6). B). CD45- cells were separated into the four stromal subsets (FRC, LEC, DN, BEC) using Podoplanin and CD31 antibodies. (TIF) [file pone.0156079.s003.tif]

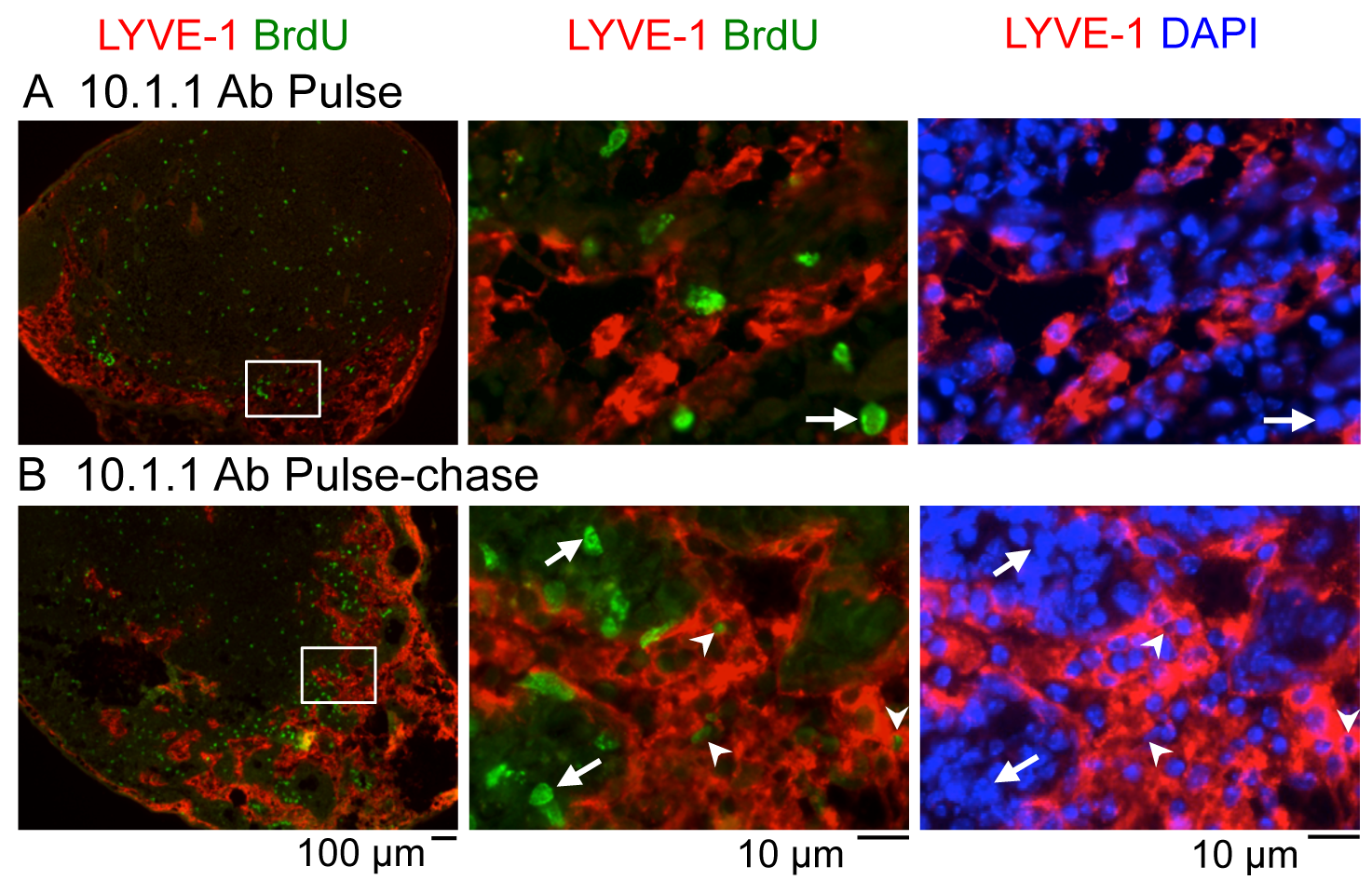

Supplement: S4 Fig — A). A second example of an LN from a pulse-labeled 10.1.1 Ab-injected mouse was immunostained with anti-LYVE-1 (red) and with anti-BrdU (green) antibodies. The LYVE-1 and BrdU-stained region outlined by the white box is shown at higher magnification in the middle panel, while the right panel shows higher magnification of the same section immunostained with LYVE-1 in combination with blue DAPI staining of nuclei. BrdU immunostaining colocalizes with DAPI nuclear staining (arrows). B). A second example of an LN from a pulse-chase-labeled mouse immunostained for LYVE-1 and BrdU. The white boxed area is shown at higher magnification in the middle panel, demonstrating increased proliferation of LECs (e.g. arrowheads) and non-LECs (e.g. arrows). The right panel shows LYVE-1 staining in combination with DAPI staining of nuclei. BrdU immunostaining colocalizes with DAPI nuclear staining in LYVE-1- non-LECs (arrows), and in LYVE-1+ LECs (arrowheads). Scale bars are indicated. (TIF) [file pone.0156079.s004.tif]
